# Technical performance of a proximity extension assay inflammation biomarker panel with synovial fluid

**DOI:** 10.1016/j.ocarto.2022.100293

**Published:** 2022-07-07

**Authors:** André Struglics, Staffan Larsson, L. Stefan Lohmander, Per Swärd

**Affiliations:** Orthopaedics, Department of Clinical Sciences Lund, Lund University, Lund, Sweden

## 1 Introduction

The plasma proteome can provide a deeper understanding of the pathophysiology of different diseases and is a key source for detection of new biomarkers. Proximity extension assay (PEA), developed by Olink Proteomics (Uppsala, Sweden), detects proteins by using pairs of antibodies that are linked to oligonucleotides; upon target binding, the probes anneal when in proximity and the oligonucleotides are extended by DNA polymerase and the newly formed antigen is quantified by real-time PCR [1]. PEA provides high specificity and sensitivity, and the possibility to measure the relative abundance of a large number of proteins (92 up to 384 biomarkers), only using a few μL of biofluid per sample. This technique makes it possible to assess low-abundant proteins which are not accessible using mass spectrometry techniques [2]. The multi-assay/plex capacity of PEA is comparable with other multiplex assays like Meso Scale Discovery (MSD); MSD uses electrochemiluminescence measuring the concentration of up to 10 biomarkers per well in 25 ​μl per sample. The PEA has an advantage over enzyme-linked immunosorbent assay (ELISA) and MSD assays in that PEA has a high number of biomarkers which are quantifiable in very low sample volume.

In plasma and serum, the correlation between the PEA technique and other techniques, including ELISA and electrochemiluminescence has been shown to be strong [[3], [4], [5], [6], [7], [8]]. The technical performance of the PEA technique in synovial fluid is unknown. The characteristics of synovial fluid differs from plasma in that some proteins are more abundant, whereas other proteins are less abundant. Compared to plasma, synovial fluid is very viscous due to high levels of hyaluronic acid [9]. These differences could interfere with the PEA analysis technique. To interpret protein data generated by the PEA technique in synovial fluid, information on the technical performance of this assay on synovial fluid samples is needed [10].

The purpose of the present study was to evaluate the technical performance of Olink's inflammation PEA with synovial fluid samples and compare it with the performance of the same PEA in the more commonly used biofluids, serum and plasma. The second aim was to compare some of the PEA data with data obtained by immunoassays using MSD.

## 2 Materials and methods

### 2.1 Samples

Synovial fluid samples were centrifuged at between 1800 and 3000×*g* for 10 ​min, the supernatant was stored at −80 ​°C until usage. The synovial fluid samples were from 756 subjects and the serum samples from 751 subjects; sample information is summarized in Table S1. The synovial fluid control samples (n ​= ​9; named A-I) were selected based on variations in tumor necrosis factor (TNF) and interleukin (IL)-6 concentrations and on levels of heme and hemolysis; one synovial fluid control sample (sample-D) was added to each synovial fluid 96-well plate (Table 1). A serum quality control sample (QC; a pool of >100 serum samples) was added to each serum 96-well plate. In addition, 14 serum samples were used as bridging samples (for comparison between experiments) and another 11 serum samples were used for inter-plate comparisons. A plasma QC sample (i.e., a pooled EDTA plasma), supplied by the PEA assay vendor, was included - added in duplicates on all 96-well plates.

**Table 1:** Synovial fluid control samples.

Subject	Diagnosis	Age, years	Sex	Hemolysis, mg/dl	Heme, μM	IL6, pg/ml	TNF, pg/ml
A	Knee injury	38	M	>1000	542	40,251	19.2
B	Knee injury	15	M	50	56.7	43,150	31.6
C	Knee injury	23	F	<20	29.8	4621	8.7
D	Knee injury	23	M	<20	26.3	1344	7.4
E	Knee injury	49	M	<20	65.7	414	5.2
F	Knee injury	18	M	20	91.7	386	4.1
G	Knee injury	17	F	>1000	221	438	6.6
H	Reference	24	M	<20	Nd	2.7	1.6
I	Reference	17	F	<20	Nd	28.2	4.8

Interleukin (IL) −6 and tumor necrosis factor (TNF) were assessed using Meso Scale discovery (MSD) immunoassay. M ​= ​male, F ​= ​female, Nd ​= ​not determined, Reference ​= ​knee-healthy subjects.

### 2.2 Heme and hemolysis

The concentration of heme in synovial fluid was determined by QuantiChrom™ Heme Assay kit (BioAssay Systems, Hayward, CA, U.S; product no. DIHM-250) following the manufacturer's instructions. Hemolysis was estimated by a visual comparison using hemolysis reference palette (Centers for Disease Control and Prevention; page last reviewed June 9, 2021).

### 2.3 Proximity extension assay (PEA)

Undiluted serum (20 μl/sample) and undiluted to 25 times diluted (in PBS) synovial fluid samples (16 μl/sample) were transferred into 96-well plates (Thermo Scientific, Waltham, Massachusetts, U.S; product no. AB-0800). The plates were covered with sealing tape (Applied Biosystem, Waltham, Massachusetts, U.S; product no. 4306311), frozen and sent to Olink Uppsala (Olink Proteomics, Uppsala Science Park, Uppsala, Sweden) for PEA analysis; Olink inflammation panel code 91301 (versions 3012, 3021 and 3022). The Olink data is expressed as normalized protein expression (NPX), an arbitrary unit on a log2-scale. Description of the PEA assessment is found as Supplementary material.

### 2.4 Cytokine assessment with Meso Scale Discovery (MSD)

IL-1β, −6, −8, −10, −12p70, interferon gamma (IFN-γ) and TNF were quantified by the Human Pro-inflammatory 7-plex immunoassay (MSD, Rockville, Maryland, U.S; product no. K15008C) in synovial fluid and serum samples as described [11], and these data were used for assessment of correlation with PEA measurements. The assessment of cytokine concentrations in the synovial fluid control samples (Table 1) has been described [12].

### 2.5 Statistics

To be able to compare the data and to easily interpret the results, the PEA data values (NPX units) were converted from log2 to linear NPX data prior to analyses. For analysis of correlation between PEA and MSD measurements, the linear data was log_10_ transformed resulting in residuals that were estimated as normally distributed based on histograms and normal probability plots, and therefore parametric Pearson's correlations were used. To maximize the statistical power in the correlation assessments, all NPX-values (above and below LLOD) were used; the PEA extrapolates NPX-values below LLOD. Only concentrations above lower limit of quantification (LLOQ) were used from the MSD assay. We used Bland-Altman plots of log_10_ transformed data to assess the level of agreement between measurements by PEA and MSD [13].

## 3 Results

The results are from four different experiments (SF-2019-04-22, SF-2019-04-23, serum-2019-04-22 and serum-2020-08-22); an experiment is herein defined as a set of samples (in 96-well plate/s) sent to Olink for PEA-analysis at the same occasion.

### 3.1 Proportion of inflammatory biomarkers

Synovial fluid and serum samples from patients with osteoarthritis (OA) and with different knee injuries and from knee healthy reference subjects were analyzed using the inflammatory PEA panel of Olink (Table S1). Approximately 98% of the 1014 synovial fluid samples and 98% of the 1656 serum samples passed the assay vendor quality control (Table 2). Of the 92 inflammatory markers (from here on referred to as biomarkers), 60 biomarkers had values above the lower limit of detection (LLOD) and were present in ≥75% of the synovial fluid samples (Table 2); similarly, 73 biomarkers with values above LLOD were present in ≥75% of the serum samples, and for the vendor plasma QC samples, 67 and 72 biomarkers (from two experiments) had values above LLOD. For one biomarker (IL-2), the concentration was below LLOD in all synovial fluid and serum samples and in the vendor QC-samples (Table 2). A complete list of the 92 biomarkers with detection rates in synovial fluid and serum samples, and in the vendor plasma QC samples, is presented as supplementary data (Table S2).

**Table 2:** The number of detected biomarkers in synovial fluid, serum and vendor plasma samples.

	Synovial fluid	Serum	Vendor plasma QC
Biomarkers:			
Detected in ≥75% of samples, n	60	73	67, 72
Detected in >90% of samples, n	50	68	67, 71
Detected in 0% of samples, n	1	1	11, 14
aExperiments, n	1	1	2
Plates, n	12	19	12, 19
Samples for analyses, n (dilution)	1014 (4x)	1656 (1x)	24 (1x), 38 (1x)
bSamples that passed QC, n (%)	998 (98.4)	1622 (97.9)	24 (100), 38 (100)
cSamples that failed, n, (%)	7 (0.7)	0 (0)	0 (0)
Sampled that did not pass QC, n (%)	9 (0.9)	34 (2.1)	0 (0)

a Experiments ​= ​analyses done at one or two different occasions at Olink.

b Samples that passed quality control (QC) of vendor and that had data values above lower limit of detection.

c Seven synovial fluid samples lacked data for all 92 biomarkers.

### 3.2 Coefficient of variation

**Fig. 1:**
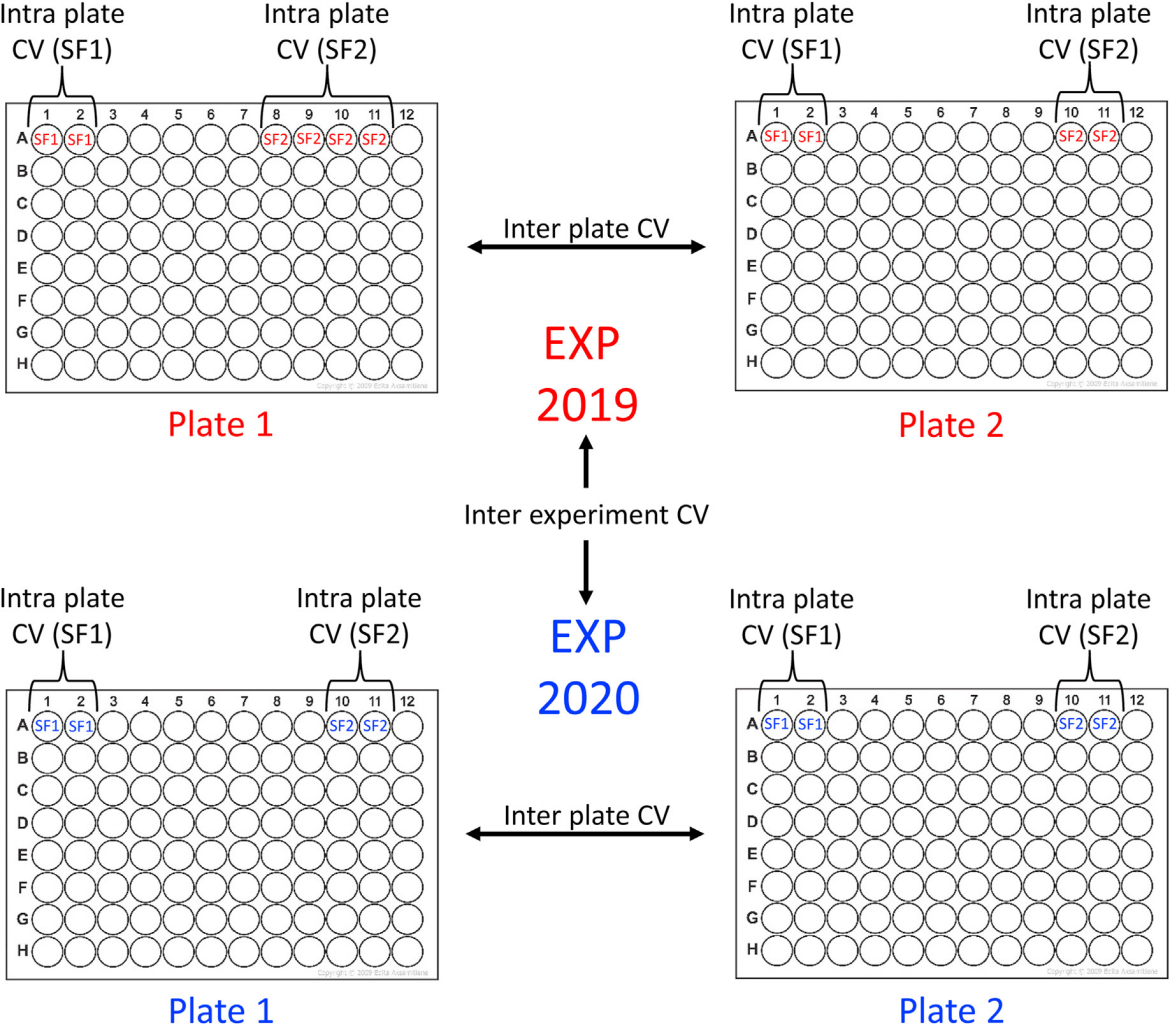
The figure illustrates intra plate assessments (here with two synovial fluid samples SF1 and SF2), inter plate assessments (here between plate 1 and 2, run in 2019 and in 2020) and an inter experiment assessment (here shown as a comparison between plates analyzed 2019 with plates analyzed 2020). CV ​= ​coefficient of variation, EXP ​= ​experiment.

The coefficient of variation (CV) was analyzed as intra plate CV (i.e. CV within 96-well plates), inter plate CV (i.e. between 96-well plates within an experiment) and as inter experiment CV (i.e. between PEA-analysis conducted at different occasions) (Fig. 1).

#### 3.2.1 Intra plate CV

The intra plate CV was analyzed in two different experiments using synovial fluid control samples (4-times diluted samples A-D; Table 1).

For the biomarkers detected in synovial fluid samples which had values above LLOD (63–72 biomarkers), the mean intra plate CV was 6.3%. Between 97 and 100% of the biomarkers (i.e. n ​= ​61–72) found in synovial fluid samples had intra CV values ​< ​20% (Table 3). Similar mean intra plate CV and amount of proteins detected were found for synovial fluid control samples which were analyzed undiluted (data not shown). Synovial fluid control sample A, which had high hemolysis and heme levels (Table 1), had similar biomarker detection rate and mean intra CV as the other synovial fluid control samples (B-D, with low to medium levels of hemolysis and heme; data not shown).

**Table 3:** Assessment of intra and inter plate coefficient of variation (CV) using synovial fluid, vendor plasma quality control (QC) and serum QC samples. Only data from biomarkers that had values above lower limit detection are presented.

		Synovial fluid	Vendor plasma QC
Intra plate	Biomarkers		
	Total, n	63–72	71–73
	Intra plate CV %, mean (range)	6.3 (0.0–25.0)	6.2 (0.0–30.1)
	With CV ​< ​20%, n (%)	61–72 (97–100)	59–72 (80–100)
	aExperiments, n	2	3
	Samples, n (dilution)	4 (4x)	1 (1x)
	Repeats/plate, n	2 or 4	2
	Plates, n	2	32

		Synovial fluid	Vendor plasma QC	Serum QC
Inter plate	Biomarkers			
	Total, n	66	67–73	78
	Inter plate CV %, mean (range)	19.1 (6.7–49.6)	13.5 (0.4–79.3)	14.8 (5.3–92.5)
	With CV ​< ​20%, n (%)	42 (64)	59–70 (81–99)	68 (87)
	Experiments, n	1	3	1
	Samples, n (dilution)	1 (4x)	1 (1x)	1 (1x)
	Repeats/plate, n	1	2	1
	Plates, n	9–12	34	15–19

a Experiments ​= ​analyses done at one, two or three different occasions at Olink.

The vendor plasma QC sample had between 71 and 73 biomarkers (from three experiments) with values above LLOD and the mean intra plate CV was 6.2%; between 80 and 100% of the biomarkers found in vendor plasma QC samples had intra CV values ​< ​20% (Table 3). A complete list of the 92 biomarkers with intra plate CV rates in the synovial fluid control samples and the assay vendor plasma QC samples is presented as supplementary data (Table S3).

#### 3.2.2 Inter plate CV

The inter plate CV was analyzed in one experiment using one synovial fluid control sample (4-times diluted sample-D; Table 1) and the serum QC sample (undiluted).

For the biomarkers detected in the synovial fluid control sample which had values above LLOD (n ​= ​66 biomarkers), the mean inter plate CV was 19.1%; 42 of the biomarkers (i.e. 64%) found in the synovial fluid sample had intra CV values ​< ​20% (Table 3). Similar inter plate CV and amount of proteins detected were found for eleven randomly selected synovial fluid samples (each sample put on two plates; data not shown).

The vendor plasma QC and serum QC samples had mean inter plate CV of 13.5% and 14.8%, respectively, and between 81 and 99% of the biomarkers found in these blood samples had intra CV values ​< ​20% (Table 3). A complete list of the 92 biomarkers with inter plate CV rates in the synovial fluid and serum QC control samples, and the vendor plasma QC sample are presented as supplementary data (Table S4).

#### 3.2.3 Inter experimental CV

The inter experimental CV was analyzed for biomarkers which had values above LLOD, and the assessment was done with or without adjustments using bridging samples (i.e. samples present in both experiments).

The biomarkers detected in the nine (A-I; Table 1) synovial fluid control samples (n ​= ​63 biomarkers found in both experiments) had a mean inter experiment CV of 33.0% (without adjustments; Table 4); no analyses were done using synovial fluid bridging sample adjustments.

**Table 4:** Assessment of inter experimental coefficient of variation (CV) using synovial fluid, serum and plasma quality control (QC) samples. The data were either adjusted (using 14 bridging samples) or unadjusted between two experiments. Only data from biomarkers that had values above lower limit of detection are presented.

	Synovial fluid	Serum		Vendor plasma QC	
	No adjustment	No adjustment	Adjusted	No adjustment	Adjusted
Biomarkers					
Total n	63	70	70	64	64
Inter experiment CV %, mean (range)	33.0 (0.1–114.6)	30.3 (0.0–134.0)	17.7 (0.0–126.3)	24.8 (0.1–130.3)	9.0 (0.1–75.5)
With CV ​< ​20%, n (%)	16 (25)	23 (33)	57 (81)	Nd	60 (95)
aExperiments, n	2	2	2	2	2
Samples, n (dilution)	9 (1x – 4x)	14 (1x)	14 (1x)	1 (1x)	1 (1x)
Repeats/plate, n	1–4	1	1	2	2
Plates per experiment, n	1 vs 7	3 vs 8	3 vs 8	3 vs 19	3 vs 19

a Experiments ​= ​analyses done at two different occasions at Olink. Nd ​= ​not determined.

The biomarkers detected in the fourteen serum control samples (n ​= ​70 biomarkers found in both experiments) had a mean inter experiment CV of 17.7% after adjustments using bridging samples; without adjustments, the mean inter experiment CV was and 30.3% (Table 4). Further, for the serum QC sample the mean inter experiment CV was after adjustments 7.8% (76 biomarkers found in both experiments; 3 vs 19 plates).

The mean inter experimental CV for the vendor plasma QC sample (n ​= ​64 biomarkers found in both experiments) was 9.0% after adjustment and 24.8% without (Table 4).

A complete list of the 92 biomarkers with inter experimental CV rates in the synovial fluid and serum control samples, and the vendor plasma QC sample, is presented as supplementary data (Table S5).

### 3.3 Dilution linearity

Dilution linearity was assessed using the nine synovial fluid control samples (A-I; Table 1), and the selection of different dilution ranges were based on the concentrations of TNF and IL-6 previously measured by the MSD immunoassay (Table 1, Table 5). The analysis was done either using *no selection criteria* (i.e. all biomarkers) or using *selection criteria* (i.e. selected biomarkers with values above LLOD, CV of linearity <21% and a recovery between 74 and 127%). In total 61 biomarkers fulfilled the selection criteria in one or more of the nine control samples (Table S6). Depending on sample groups (A-D, E-G or H, I) and which dilution range within the sample group was used, the number of biomarkers fulfilling the selection criteria were between 19 and 51 with a mean linearity CV between 10.5 and 14% (CV range ​= ​1.7–20.8%) (Table 5, Table S7). Similarly, the mean linearity CV for the assessment not using the selection criteria was between 5.2 and 43.7% (CV range ​= ​1.7–358.2%) (Table 5).

**Table 5:** Assessment of dilution linearity recovery of the nine synovial fluid control samples (A-I). The analyses were done with or without predefined biomarker selection criteria at different dilution ranges. The data presented in this table was from one experiment (plate) where the samples were diluted and assessed as single sample up to quadruplicates.

		Samples A-D		Samples E-G		Samples H,I
aSelection criteria	Dilutions, range (span)	1–25x (25x)	2–25x (12.5x)	1–10x (10x)	2–10x (5x)	1–4x (4x)
	Sample dilutions	1x, 2x, 4x, 10x, 25x	2x, 4x, 10x, 25x	1x, 2x, 4x, 10x	2x, 4x, 10x	1x, 2x, 4x
	Samples, n	4	4	3	3	2
	Biomarkers					
	N^b^	19–36	32–41	42–48	42–51	44
	CV % of NPX, mean (range)	11.8 (1.7–20.0)	10.5 (1.3–20.1)	10.9 (2.5–20.2)	10.9 (2.1–19.7)	14.0 (6.4–20.8)
	Range of recovery, %	74.4–126.4	75.5–125.8	75.1–126.0	77.5–120.9	76.7–123.2
		Samples A-D		Samples E-G		Samples H, I
No selection criteria	Dilutions, range (span)	1–25x (25x)	2–25x (12.5x)	1–10x (10x)	2–10x (5x)	1–4x (4x)
	Sample dilution, times	1x, 2x, 4x, 10x, 25x	2x, 4x, 10x, 25x	1x, 2x, 4x, 10x	2x, 4x, 10x	1x, 2x, 4x
	Samples, n	4	4	3	3	2
	Biomarkers					
	All, n	92	92	92	92	92
	Values ​< ​LLOD, n^c^	17–27	17–27	22	22	19, 26
	CV % of NPX, mean (range)	5.2 (1.7–145.5)	43.7 (1.7–145.5)	37.8 (2.5–117)	33.4 (2.5–117)	31.7 (6.4–82.2)
	Range of recovery, %	8.6–358.2	14.2–298.7	10.9–272.6	26.5–216.5	26.4–188.7

NPX ​= ​normalized protein expression.

a Selection criteria ​= ​values above lower limit of detection (LLOD), coefficient of variation (CV) of linearity <21% and recovery between 74 and 127%.

b N ​= ​the range of number of biomarkers detected in sample groups at different dilutions. In Supplementary Table S7 the number of biomarkers detected in each sample (A-I) are presented.

c Number of biomarker with values < LLOD in individual samples (A-I): 17 (A), 20 (B), 27 (C), 18 (D), 22 (E, F, G), 26 (H) and 19 (I).

### 3.4 Temperature and freeze-thaw treatments

Two or three synovial fluid control samples (C-E; Table 1), were used to assess the influence of temperature and freeze-thaw treatments on biomarkers. The samples were analyzed either undiluted or diluted 4-times (Table 6). Depending on sample and dilution, between 63 and 78 biomarkers had values above LLOD and were included in these assessments. The effect of temperature or freeze-thaw treatment of synovial fluid control samples on all 92 biomarkers are presented as recovery of NPX (Tables S8 and S9).

**Table 6:** Effect of temperature and freeze-thaw treatments on biomarkers in synovial fluid samples. Ratio of NPX-values between temperature or freeze-thaw treated samples vs control (no treatment) are shown. The results are from one experiment and only data from biomarkers that had values above lower limit of quantification are presented.

	2 ​h at room temp.		24 ​h at 4 ​°C	
	Undiluted	4x dilution	Undiluted	4x dilution
Samples for analyses, n	3	2	3	2
Biomarkers, n	70, 76, 78	64, 71	71, 77, 77	64, 73
NPX ratio, mean (range)	1.03 (0.81–1.72)	0.99 (0.77–1.28)	0.95 (0.61–1.36)	1.05 (0.81–1.65)
Biomarkers with NPX ratio between 0.8 and 1.2, n (%)	67, 72, 76 (96–97)	63, 69 (98, 97)	66, 69, 73 (93–95)	61, 68 (95, 93)
	3 cycles		10 cycles	
	Undiluted	4x dilution	Undiluted	4x dilution
Samples for analyses, n	3	2	2	3
Biomarkers, n	69, 77, 78	65, 71	70, 77	63, 70, 72
NPX ratio, mean (range)	1.00 (0.73–1.35)	1.07 (0.85–1.37)	1.02 (0.83–1.20)	0.97 (0.70–1.30)
Biomarkers with NPX ratio between 0.8 and 1.2, n (%)	68, 74, 76 (97–99)	60, 71 (92, 100)	70, 77 (100, 100)	61, 69, 70 (97–99)

NPX ​= ​normalized protein expression.

#### 3.4.1 Temperature treatment

The mean ratio (treatment divided by control) of biomarker NPX-values between the *2h room temperature* treatment and control were 1.03 for the undiluted samples and 0.99 for the 4-times diluted samples; similarly, the mean ratio between the *24h at 4*^*o*^*C treatment* and control were 0.95 for the undiluted samples and 1.05 for the 4-times diluted samples (Table 6). The number of biomarkers that had NPX ratios between 0.8 and 1.2 were 61–76 (depending on samples, dilutions and temperature treatment), which corresponds to 93–98% of the biomarkers (Table 6). Sample D had only seven biomarkers with NPX-ratios between 0.8 and 1.2 (i.e. for *2h room temperature*), and sample E had only 18 biomarkers with NPX-ratios between 0.8 and 1.2 (i.e., for *24h at 4*^*o*^*C*); due to low number of detected biomarkers these samples were considered as outliers and were not included in the results presented in Table 6.

#### 3.4.2 Freeze-thaw treatment

The mean ratio of biomarker NPX-values between the *three freeze-thaw cycle* treatment and control were 1.00 for the undiluted samples and 1.07 for the 4-times diluted samples; similarly, the mean ratio between the *ten freeze-thaw cycles* and control were 1.02 for the undiluted samples and 0.97 for the 4-times diluted samples (Table 6). The number of biomarkers that had NPX ratios between 0.8 and 1.2 were 60–77 depending on samples and dilutions (depending on samples, dilutions and temperature treatment), which corresponds to 92–100% of the biomarkers (Table 6).

### 3.5 Association between PEA and MSD measurements

From the knee injury group (Table S1), 310 synovial fluid and 546 serum samples were used for assessments of associations between the PEA and MSD measurement. Of the MSD 7-plex, five cytokines (IL-6, IL-8, IL-10, IFN-γ and TNF) have been evaluated previously [11] and were used in this comparison.

#### 3.5.1 Synovial fluid

**Fig. 2:**
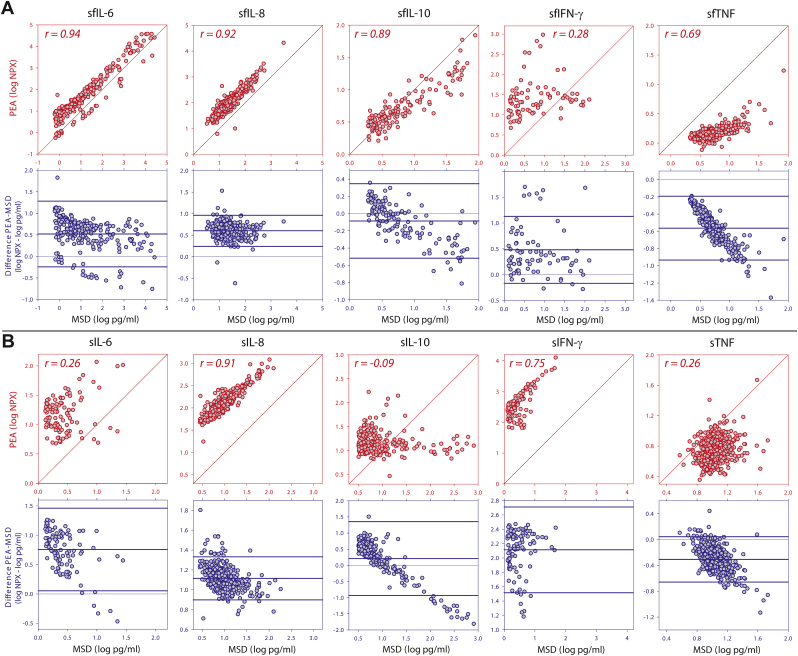
Bivariate scatterplots of the relationship between measurements by Meso Scale Discovery (MSD) and proximity extension assay (PEA) in synovial fluid (A) and serum (B). For each panel two graphs are shown: *Above in red*, log transformed cytokine concentration measured by MSD and PEA with a diagonal line of equality and the Pearson correlation coefficient (r). *Below in blue*, Bland-Altman plots of difference versus MSD measurement after log transformation with mean difference and 95% limits of agreements as thick horizontal lines, with a thin line at 0 indicating no difference. The statistical data for the correlation analyses are shown in Table S10. NPX ​= ​normalized protein expression. s ​= ​serum. sf ​= ​synovial fluid.

In synovial fluid, all five cytokines were positively correlated between the two measurements with the lowest correlation coefficient (r) of 0.28 for IFN-γ and the highest for IL-6 (r ​= ​0.94) (Fig. 2A, Table S10). A sensitivity analysis (only using NPX-values above LLOD) resulted in marginal differences for all cytokines, but for INF-γ the correlation between assays was no longer statistically significant (r ​= ​0.06, P ​= ​0.66, n ​= ​57). In the analysis of agreement between assay measurements in synovial fluid, the bivariate scatter and Bland-Altman plots revealed that only IL-8 had narrow limits of agreement and no proportional bias between measurements in the two assays (Fig. 2A, Table S11). The estimated effect of the proportional bias on the difference between groups with high and low concentrations was between 1.0 (IL-8) and 4.9 (IL-6) times smaller when measured with PEA compared to when measured with MSD (Table S11).

#### 3.5.2 Serum

In serum, four cytokines were positively correlated between measurements, with the lowest correlation coefficient for IL-6 and TNF (both r ​= ​0.26) and the highest for IL-8 (r ​= ​0.91); for serum IL-10, there was no correlation between the assays (Fig. 2B, Table S10). The sensitivity analysis showed similar results (data not shown). In the analysis of agreement between assay measurements in serum, only IFN-γ had narrow limits of agreement and no proportional bias between measurements in the two assays (Fig. 2B, Table S11). The most extreme difference between assays was seen for IL-10, where there was no difference between the groups with high and low concentrations when measured with PEA, compared to a 77-fold difference in means when measured with MSD (Table S11). For the other three serum cytokines (IL-6, IL-8 and TNF) the estimated effect of the proportional bias on the difference between groups with high and low concentrations was between 1.6 and 3.6 times smaller when measured with PEA compared to when measured with MSD (Table S11).

## 4 Discussion

A large set of synovial fluid (n ​= ​1014) and serum (n ​= ​1656) samples from patients with knee OA, knee injury and knee healthy subjects were assessed in this study. Fewer biomarkers, that passed the PEA quality control and had values above LLOD, were found in the synovial fluid samples compared to serum and vendor plasma QC samples. The intra plate CV for synovial fluid samples were similar to the CV from blood samples (mean CV around 6%), while the inter plate CV was a slightly higher (based on the mean CV).

Synovial fluid has not been validated as a biofluid/sample for PEA. To support conclusions based on PEA-data from measurements of synovial fluid samples, a validation of the technical performance of this assay/technique using synovial fluid is necessary. We also present the technical performance for each of the 92 inflammatory biomarkers (Supplementary data), making it possible to evaluate a specific biomarker of interest.

Based on Olink's analysis certificates for the inflammation panel, using EDTA-plasma samples from healthy donors, more than 69 biomarkers are detectable in more than 75% of the samples. This level of detection was achieved for our serum samples and for the Vendor plasma QC sample, but for the synovial fluid samples only 60 biomarkers were detectable in more than 75% of the samples.

The intra and inter CV for ligand-binding assays are recommended to be below 20% [10,14]. According to the Olink validation data of inflammation panel [15], the mean intra CV for plasma samples (n ​= ​7) was 7% (range 5–14%) for the 92 inflammatory markers; for the same markers and plasma samples, the mean inter CV was 19% (range 8–31%). The synovial fluid intra plate CV was in the same range as the vendor plasma QC, although the inter plate CV was higher, with only 42 biomarkers having inter CV below 20%.

The inter experiment CV without bridging sample adjustments are quite high (mean CV between 25 and 33%, depending on biofluid), but after adjustments these CV values decrease and are more similar to inter plate (within experiment) CV values (i.e. between 10 and 20%, for serum and plasma QC).

The dilution linearity is recommended to be +/− ≤ 20% of the expected values in an immunoassay [10,14]. To capture as many biomarkers as possible, we set the selection criteria in this study as a recovery between 74 and 127% with a CV of linearity <21%. Considering the low number of biomarkers that fulfilled this selection criteria (n ​= ​19–51) in the dilution series of synovial fluid samples, it might be better to use a fixed dilution of the synovial fluid samples (e.g. 4-times dilution in PBS as was the default dilution of synovial fluid samples in this study).

Synovial fluid has approximately 10–40 times higher viscosity compared to serum and/or plasma [9]. To ease the handling of the synovial fluid samples at the vendor site, it would therefore be a good idea to dilute these samples. Considering the large dilution range of the PEA, approximately from 500 to 400 ​000 (based on concentration values for 90 biomarkers of the PEA inflammation panel), a fixed dilution should capture most of the biomarkers in the synovial fluid samples. This was the case for the 1014 synovial fluid samples where 60 biomarkers were detected in more than 75% samples tested.

The recommended stability recovery for immunoassays is ​± ​20% of the expected values [10,16]. When this recommendation was applied for our stability tests of synovial fluid samples (storage at different temperatures and freeze-thaw cycles), it resulted in a high percentage (92–100%) of biomarkers (i.e. immunoassays of biomarkers) that passed the recommended stability recovery. The conclusion we can draw from these stability tests is that the PEA inflammation panel of 92 biomarkers does not seem to be especially sensitive to freeze-thaw and temperature treatments of synovial fluid samples.

Using plasma or serum samples from different patient groups, several groups have validated the PEA (different biomarker panels) by comparison (association analyses) against conventional immunoassays (ELISA and electrochemiluminescence) [[3], [4], [5], [6],^17^], or against mass spectrometry [2,18]. For the synovial fluid samples in this study, there was a moderate to very strong correlation (according to Schober et al. definition of strength of linear relationship [19]) between the PEA and MSD measurements for IL-6, IL-8, IL-10 and TNF, while for IFN-γ the correlation was weak. For serum in this study, a similar strong/very strong correlation was only found for IL-8 and IFN-γ. In concordance, a strong correlation between ELISA and PEA measurements for IL-8 in EDTA-plasma has been shown [3]. The serum IL-10 data showed no correlation between PEA and MSD measurements in this study. This could in some part be explained by the low abundance of IL-10 in our serum samples (46% of the IL-10 serum data had values above LLOQ of the MSD assay [11]) and was thereby more difficult to assess by the MSD assay, giving a narrow distribution around the mean. A similar explanation was proposed [2], although the large proportion of samples excluded due to low concentrations may partly explain the absence of correlation between MSD and PEA measurements of serum IL-10. For synovial fluid IL-10, measured within a similar concentration span as in serum, there was a very strong correlation between measurements in PEA and MSD. This complete discordance between the very strong correlation between assay measurements of IL-10 in synovial fluid an absence of correlation in serum suggests that there is a difference in detection of IL-10 between serum and synovial fluid in the PEA assay, where the recognition and measurement in synovial fluid is more similar to the conventional ELISA format in MSD than in serum (Table S11).

We used nine synovial fluid control samples from knee-healthy and knee injured subjects (Table 1) in the different validations of the PEA inflammation panel, but not all control samples were used in all validations. For instance, in the validation of dilution linearity only 2 to 4 control samples per dilution group (4-, 5-, 10-, 12.5- or 25-times) were used. A larger set of synovial fluid control samples from the same subject groups or samples from other patient groups (e.g., rheumatoid arthritis) could have generated more consistent dilution linearity data with additional biomarkers fulfilling the selection criteria (Table 5). Further, serum samples were only used in some of the validations of PEA, and if we had analyzed dilution linearity and treatment effects (freeze-thaw cycles and temperature) also on serum samples, then a more complete comparison between the two biofluids would have been possible. We did not examine the effect of hyaluronidase treatment of synovial fluid samples, which might have facilitated the detection of some of the less abundant biomarkers and reduced possible matrix effects.

Strengths of our study include: (i) a large set of synovial fluid samples with low (i.e. knee healthy reference samples) to high (i.e. OA and knee injury samples) inflammation biomarker concentrations; (ii) validation of the PEA inflammation panel with synovial fluid samples was compared with validation of serum and plasma samples.

In summary, we found fewer biomarkers that passed the PEA quality control and had values above LLOD in the synovial fluid samples, compared to serum and vendor plasma QC samples. The intra plate CVs for synovial fluid samples were similar to the CVs from blood samples, while the inter plate CV was slightly higher. Although the performance of the PEA technology in synovial fluid samples was overall satisfying, there were some concerns regarding the dilution linearity of several of the assessed cytokines. This could be related to matrix effects, as well as the low abundancy of certain cytokines in the assessed samples. Based on the data in this study, we suggest diluting synovial fluid 4 times, but avoiding use of different dilutions. Overall, we suggest that the PEA inflammation panel is equally well suited for synovial fluid samples as for the more commonly assessed serum and plasma samples.

## Ethical statement

All subjects consented to take part in this study, which was approved by the regional ethical review board at Lund University.

## Author contributions

All authors (AS, SLa, SLo, PS) contributed in designing the study. AS, SLa and PS analyzed the data and all authors (AS, SLa, SLo, PS) contributed in writing the manuscript and approved the final version.

## Role of the funding source

The study was funded by: the 10.13039/501100004359Swedish Research Council, Sweden; 10.13039/501100003173Crafoord Foundation, Sweden; the 10.13039/501100007949Swedish Rheumatism Association, Sweden; the 10.13039/501100006738Faculty of Medicine at Lund University, Sweden; Region Skåne Governmental funding of clinical research within the national health services (10.13039/501100005390ALF), Sweden; Kocks Foundation, Sweden; 10.13039/501100005390Alfred Österlund Foundation, Sweden.

## Declaration of competing interest

All authors declare that they have no competing interests.

## References

[1] Assarsson E., Lundberg M., Holmquist G., Bjorkesten J., Thorsen S.B., Ekman D. (2014). Homogenous 96-plex PEA immunoassay exhibiting high sensitivity, specificity, and excellent scalability. PLoS One.

[2] Petrera A., von Toerne C., Behler J., Huth C., Thorand B., Hilgendorff A. (2021). Multiplatform approach for plasma Proteomics: complementarity of Olink proximity extension assay technology to mass spectrometry-based protein profiling. J. Proteome Res..

[3] Thorsen S.B., Lundberg M., Villablanca A., Christensen S.L., Belling K.C., Nielsen B.S. (2013). Detection of serological biomarkers by proximity extension assay for detection of colorectal neoplasias in symptomatic individuals. J. Transl. Med..

[4] Siegbahn A., Eriksson N., Lindbäck J., Wallentin L. (2017). Advancing Precision Medicine: Current and Future Proteogenomic Strategies for Biomarker Discovery and Development.

[5] Graumann J., Finkernagel F., Reinartz S., Stief T., Brodje D., Renz H. (2019). Multi-platform affinity Proteomics identify proteins linked to metastasis and immune suppression in ovarian cancer plasma. Front. Oncol..

[6] Sama I.E., Ravera A., Santema B.T., van Goor H., Ter Maaten J.M., Cleland J.G.F. (2020). Circulating plasma concentrations of angiotensin-converting enzyme 2 in men and women with heart failure and effects of renin-angiotensin-aldosterone inhibitors. Eur. Heart J..

[7] Olink White Paper (2021). https://www.olink.com/content/uploads/2021/09/olink-white-paper-a-comparative-study-across-multiple-platforms-v1.2.pdf.

[8] Olink White Paper (2021). https://www.olink.com/content/uploads/2021/09/olink-technical-comparisons-and-orthogonal-validation-1118-v2.0.pdf.

[9] Diem K., Lentner C. (1970).

[10] DeSilva B., Smith W., Weiner R., Kelley M., Smolec J., Lee B. (2003). Recommendations for the bioanalytical method validation of ligand-binding assays to support pharmacokinetic assessments of macromolecules. Pharmaceut. Res..

[11] Struglics A., Larsson S., Kumahashi N., Frobell R., Lohmander L.S. (2015). Changes in cytokines and aggrecan ARGS neoepitope in synovial fluid and serum and in C-terminal crosslinking telopeptide of type II collagen and N-terminal crosslinking telopeptide of type I collagen in urine over five years after anterior cruciate ligament rupture: an exploratory analysis in the knee anterior cruciate ligament, nonsurgical versus surgical treatment trial. Arthritis Rheumatol..

[12] Swärd P., Frobell R., Englund M., Roos H., Struglics A. (2012). Cartilage and bone markers and inflammatory cytokines are increased in synovial fluid in the acute phase of knee injury (hemarthrosis)--a cross-sectional analysis. Osteoarthritis and cartilage/OARS. Osteoarthr. Res. Soc..

[13] Bland J.M., Altman D.G. (1999). Measuring agreement in method comparison studies. Stat. Methods Med. Res..

[14] Smolec J., DeSilva B., Smith W., Weiner R., Kelly M., Lee B. (2005). Bioanalytical method validation for macromolecules in support of pharmacokinetic studies. Pharmaceut. Res..

[15] Olink White Paper: Olink - Inflammation. Article number 95302 (0993, v3.0, 2019-03-29): https://www.olink.com/content/uploads/2021/09/olink-inflammation-validation-data-v3.0.pdf (accessed 12 August 2021).

[16] van de Merbel N., Savoie N., Yadav M., Ohtsu Y., White J., Riccio M.F. (2014). Stability: recommendation for best practices and harmonization from the global bioanalysis consortium harmonization team. AAPS J..

[17] Sundberg I., Rasmusson A.J., Ramklint M., Just D., Ekselius L., Cunningham J.L. (2020). Daytime melatonin levels in saliva are associated with inflammatory markers and anxiety disorders. Psychoneuroendocrinology.

[18] Ali N., Turkiewicz A., Hughes V., Folkesson E., Tjornstand J., Neuman P. (2022). Proteomics profiling of human synovial fluid suggests increased protein interplay in early-osteoarthritis (OA) that is lost in late-stage OA. Mol. Cell Proteomics.

[19] Schober P., Boer C., Schwarte L.A. (2018). Correlation coefficients: appropriate use and interpretation. Anesth. Analg..

