# Supplementary material for: Technical performance of a proximity extension assay inflammation biomarker panel with synovial fluid
Source: Osteoarthr Cartil Open. 2022 Jul 7;4(3):100293. doi: 10.1016/j.ocarto.2022.100293 (PMC9718077; doi:10.1016/j.ocarto.2022.100293)
Supplement: Multimedia component 1 [file mmc1.docx]

Technical performance of a proximity extension assay inflammation biomarker panel with synovial fluid

André Struglics, PhD^†^*, Staffan Larsson, PhD^†^, L. Stefan Lohmander, MD, PhD^†^, Per Swärd MD, PhD^†^

† Orthopaedics, Department of Clinical Sciences Lund, Lund University, Lund, Sweden

**Corresponding author and reprint requests:*

André Struglics*,* Lund University, Faculty of Medicine, Department of Clinical Sciences Lund, Orthopaedics, BMC C12, Klinikgatan 28, SE-221 84 Lund, Sweden; Tel +46 46 222 0762; andre.struglics@med.lu.se.

Supplemental documents

**MATERIALS AND METHODS**

**Proximity extension assay (PEA)**

The Olink PEA enables multiplex analysis of a large number of plasma proteins (around 1400). We here assessed the Olink inflammation panel, which measures 92 proteins (Table S2). Firstly, using this technique there is an immunoreaction with monoclonal or polyclonal antibodies (PEA probes). Because the target proteins are bound in a pair-wise manner, all cross-reactive events should be prevented. This is followed by a nucleotide extension part where the oligonucleotides come in close proximity and hybridize, generating a unique DNA-sequence used for digital identification of the specific protein assay. Thereafter, qPCR is performed to amplify, detect, and enable a readout. In the assay, four internal spike-in controls (in each well), a negative control (buffer, in three wells), an external control (pooled vendor EDTA plasma sample, in two wells) and an inter plate control (for normalization, in three wells) are integrated and monitored [1, 2]. Data is presented as Normalized Protein eXpression (NPX) values, which is an arbitrary unit on a log2-scale (Olink White Paper 1096). The lower limit of detection (LLOD) is defined by Olink as the mean NPX-value of the negative control plus 3 standard deviations. The results (expressed as NPX) are logarithmically related to absolute protein concentrations, and therefore any interpretations are relative and not absolute.

REFERENCES

1. Assarsson E, Lundberg M, Holmquist G, Bjorkesten J, Thorsen SB, Ekman D, et al. Homogenous 96-plex PEA immunoassay exhibiting high sensitivity, specificity, and excellent scalability. PloS one. 2014;9(4):e95192.
2. Olink White paper: Data normalization and standardization. 1096 (v2.0, 2021-04-08): <https://www.olink.com/content/uploads/2021/09/olink-data-normalization-white-paper-v2.0.pdf> (accessed 12 August 2021).

| **Table S1**. Summary of the numbers of experiments and samples used. | | | |  |
| --- | --- | --- | --- | --- |
|  |  | Synovial fluid | Serum | |
| **All samples** |  |  |  | |
| ^1^Experiments, n |  | 2 | 2 | |
| Total 96-well plates, n |  | 13 | 22 | |
| Total samples, n |  | 1102 | 1828 | |
| Subjects, n |  | 756 | 751 | |
| **Samples per diagnostic group** |  |  |  | |
| ^2^Reference, n |  | 107 | 181 | |
| Knee injury, n |  | 732 | 1288 | |
| Osteoarthritis, n |  | 263 | 359 | |
| ^1^Experiments = analyses done at four different occasions at Olink.  ^2^Reference = samples from knee-healthy subjects. | | | |  |

**Table S2**. Data values above lower limit of detection (LLOD) in synovial fluid (total n = 1014) and serum (total n = 1656) samples, and in the Olink (vendor) plasma quality control (QC) sample (total n = 24; from one experiment).

| **Biomarkers from the Olink inflammation panel (n = 92)** | | **Data values above LLOD per total data, %** | | |
| --- | --- | --- | --- | --- |
| Proteins (abbreviated protein or gene names) | UniProt no | Synovial fluid | Serum | Vendor plasma QC |
| Adenosine Deaminase (ADA) | P00813 | 95.33 | 97.95 | 100.0 |
| Artemin (ARTN) | Q5T4W7 | 4.17 | 26.45 | 0.0 |
| Axin-1 (AXIN1) | O15169 | 78.45 | 80.56 | 0.0 |
| Beta-nerve growth factor (Beta-NGF) | P01138 | 38.23 | 97.58 | 100.0 |
| Caspase 8 (CASP-8 ) | Q14790 | 99.01 | 97.89 | 50.0 |
| C-C motif chemokine 4 (CCL4 ) | P13236 | 99.01 | 97.95 | 100.0 |
| C-C motif chemokine 19 (CCL19) | Q99731 | 98.81 | 97.95 | 100.0 |
| C-C motif chemokine 20 (CCL20) | P78556 | 85.20 | 97.95 | 100.0 |
| C-C motif chemokine 23 (CCL23) | P55773 | 99.11 | 97.95 | 100.0 |
| C-C motif chemokine 25 (CCL25) | O15444 | 87.29 | 97.95 | 100.0 |
| C-C motif chemokine 28 (CCL28) | Q9NRJ3 | 99.01 | 97.95 | 100.0 |
| CD40L receptor (CD40) | P25942 | 99.11 | 97.95 | 100.0 |
| CUB domain-containing protein 1 (CDCP1) | Q9H5V8 | 98.81 | 97.89 | 100.0 |
| C-X-C motif chemokine 1 (CXCL1) | P09341 | 99.01 | 97.95 | 100.0 |
| C-X-C motif chemokine 5 (CXCL5) | P42830 | 94.94 | 97.95 | 100.0 |
| C-X-C motif chemokine 6 (CXCL6) | P80162 | 98.81 | 97.95 | 100.0 |
| C-X-C motif chemokine 9 (CXCL9 ) | Q07325 | 99.11 | 97.95 | 100.0 |
| C-X-C motif chemokine 10 (CXCL10) | P02778 | 99.01 | 97.95 | 100.0 |
| C-X-C motif chemokine 11 (CXCL11) | O14625 | 88.48 | 97.95 | 100.0 |
| Cystatin D (CST5) | P28325 | 99.11 | 97.95 | 100.0 |
| Delta and Notch-like epidermal growth factor-related receptor (DNER) | Q8NFT8 | 99.11 | 97.95 | 100.0 |
| Eotaxin-1 (CCL11) | P51671 | 98.71 | 97.95 | 100.0 |
| Eukaryotic translation initiation factor 4E-binding protein 1 (4E-BP1) | Q13541 | 98.11 | 87.50 | 100.0 |
| Fibroblast growth factor 5 (FGF-5) | P12034 | 4.07 | 1.39 | 0.0 |
| Fibroblast growth factor 19 (FGF-19) | O95750 | 98.91 | 97.95 | 100.0 |
| Fibroblast growth factor 21 (FGF-21) | Q9NSA1 | 66.34 | 87.50 | 100.0 |
| Fibroblast growth factor 23 (FGF-23) | Q9GZV9 | 7.06 | 1.69 | 95.8 |
| Fms-related tyrosine kinase 3 ligand (Flt3L) | P49771 | 99.11 | 97.95 | 100.0 |
| Fractalkine (CX3CL1 ) | P78423 | 98.41 | 97.95 | 100.0 |
| Glial cell line-derived neurotrophic factor (GDNF) | P39905 | 1.29 | 81.76 | 100.0 |
| Hepatocyte growth factor (HGF) | P14210 | 99.11 | 97.95 | 100.0 |
| Interferon gamma (IFN-gamma) | P01579 | 60.32 | 98.55 | 95.8 |
| Interleukin-1 alpha (IL-1 alpha) | P01583 | 7.35 | 8.33 | 0.0 |
| Interleukin-2 (IL-2) | P60568 | 0.0 | 0.0 | 0.0 |
| Interleukin-2 receptor subunit beta (IL-2RB) | P14784 | 0.40 | 65.76 | 0.0 |
| Interleukin-4 (IL-4) | P05112 | 5.16 | 33.33 | 100.0 |
| Interleukin-5 (IL-5) | P05113 | 1.99 | 41.12 | 100.0 |
| Interleukin-6 (IL-6) | P05231 | 81.63 | 97.64 | 75.0 |
| Interleukin-7 (IL-7) | P13232 | 85.80 | 97.95 | 100.0 |
| Interleukin-8 (IL-8) | P10145 | 99.11 | 97.95 | 100.0 |
| Interleukin-10 (IL-10) | P22301 | 42.40 | 97.64 | 100.0 |
| Interleukin-10 receptor subunit alpha (IL-10RA) | Q13651 | 8.54 | 66.12 | 29.2 |
| Interleukin-10 receptor subunit beta (IL-10RB) | Q08334 | 99.01 | 97.95 | 100.0 |
| Interleukin-12 subunit beta (IL-12B) | P29460 | 99.11 | 97.95 | 100.0 |
| Interleukin-13 (IL-13) | P35225 | 3.67 | 24.21 | 100.0 |
| Interleukin-15 receptor subunit alpha (IL-15RA) | Q13261 | 7.94 | 92.09 | 45.8 |
| Interleukin-17A (IL-17A) | Q16552 | 25.77 | 91.12 | 100.0 |
| Interleukin-17C (IL-17C) | Q9P0M4 | 0.50 | 15.76 | 62.5 |
| Interleukin-18 (IL-18) | Q14116 | 99.11 | 97.95 | 100.0 |
| Interleukin-18 receptor 1 (IL-18R1) | Q13478 | 98.81 | 97.95 | 100.0 |
| Interleukin-20 (IL-20) | Q9NYY1 | 0.89 | 3.99 | 0.0 |
| Interleukin-20 receptor subunit alpha (IL-20RA) | Q9UHF4 | 2.38 | 27.60 | 25.0 |
| Interleukin-22 receptor subunit alpha-1 (IL-22 RA1) | Q8N6P7 | 0.70 | 11.47 | 0.0 |
| Interleukin-24 (IL-24) | Q13007 | 4.58 | 14.94 | 4.2 |
| Interleukin-33 (IL-33) | O95760 | 7.55 | 3.26 | 0.0 |
| Latency-associated peptide transforming growth factor beta 1 (LAP TGF-beta-1) | P01137 | 99.01 | 97.95 | 100.0 |
| Leukemia inhibitory factor (LIF) | P15018 | 75.17 | 10.93 | 0.0 |
| Leukemia inhibitory factor receptor (LIF-R) | P42702 | 69.12 | 97.89 | 100.0 |
| Macrophage colony-stimulating factor 1 (CSF-1) | P09603 | 99.11 | 97.95 | 100.0 |
| Macrophage inflammatory protein 1-alpha (CCL3) | P10147 | 99.11 | 97.95 | 100.0 |
| Matrix metalloproteinase-1 (MMP-1) | P03956 | 99.01 | 97.95 | 100.0 |
| Matrix metalloproteinase-10 (MMP-10) | P09238 | 98.91 | 97.95 | 100.0 |
| Monocyte chemotactic protein 1 (MCP-1) | P13500 | 99.11 | 97.95 | 100.0 |
| Monocyte chemotactic protein 2 (MCP-2) | P80075 | 99.11 | 97.95 | 100.0 |
| Monocyte chemotactic protein 3 (MCP-3) | P80098 | 52.63 | 90.28 | 0.0 |
| Monocyte chemotactic protein 4 (MCP-4) | Q99616 | 99.11 | 97.95 | 100.0 |
| Natural killer cell receptor 2B4 (CD244) | Q9BZW8 | 99.01 | 97.95 | 100.0 |
| Neurotrophin-3 (NT-3) | P20783 | 4.07 | 94.50 | 100.0 |
| Neurturin (NRTN) | Q99748 | 1.99 | 20.29 | 91.7 |
| Oncostatin-M (OSM) | P13725 | 85.30 | 97.95 | 100.0 |
| Osteoprotegerin (OPG) | O00300 | 99.11 | 97.95 | 100.0 |
| Programmed cell death 1 ligand 1 (PD-L1) | Q9NZQ7 | 98.51 | 97.95 | 100.0 |
| Protein S100-A12 (EN-RAGE ) | P80511 | 65.64 | 97.95 | 100.0 |
| Signaling lymphocytic activation molecule (SLAMF1) | Q13291 | 5.56 | 97.28 | 100.0 |
| SIR2-like protein 2 (SIRT2) | Q8IXJ6 | 65.94 | 89.86 | 0.0 |
| STAM-binding protein (STAMPB) | O95630 | 98.91 | 97.95 | 100.0 |
| Stem cell factor (SCF) | P21583 | 99.11 | 97.95 | 100.0 |
| Sulfotransferase 1A1 (ST1A1) | P50225 | 98.21 | 92.45 | 0.0 |
| T-cell surface glycoprotein CD5 (CD5) | P06127 | 98.81 | 97.95 | 100.0 |
| T-cell surface glycoprotein CD6 isoform (CD6) | P30203 | 94.44 | 97.95 | 100.0 |
| T-cell surface glycoprotein CD8 alpha chain (CD8A) | P01732 | 99.11 | 97.95 | 100.0 |
| Thymic stromal lymphopoietin (TSLP) | Q969D9 | 1.29 | 7.73 | 0.0 |
| TNF-beta (TNFB) | P01374 | 93.84 | 97.95 | 100.0 |
| TNF-related activation-induced cytokine (TRANCE) | O14788 | 76.76 | 97.95 | 100.0 |
| TNF-related apoptosis-inducing ligand (TRAIL) | P50591 | 98.91 | 97.95 | 100.0 |
| Transforming growth factor alpha (TGF-alpha) | P01135 | 98.71 | 97.95 | 100.0 |
| Tumor necrosis factor (Ligand) superfamily, member 12 (TWEAK) | O43508 | 99.11 | 97.95 | 100.0 |
| Tumor necrosis factor (TNF) | P01375 | 78.66 | 97.52 | 95.8 |
| Tumor necrosis factor ligand superfamily member 14 (TNFSF14) | O43557 | 61.77 | 97.83 | 100.0 |
| Tumor necrosis factor receptor superfamily member 9 (TNFRSF9) | Q07011 | 99.11 | 97.95 | 100.0 |
| Urokinase-type plasminogen activator (uPA) | P00749 | 99.11 | 97.95 | 100.0 |
| Vascular endothelial growth factor A (VEGF-A) | P15692 | 99.11 | 97.95 | 100.0 |

**Table S3**. Assessment of intra plate coefficient of variation (CV) using five synovial fluid control samples (each diluted 4 times) with 2 or 4 repeats per sample, and the Olink (vendor) plasma quality control (QC) sample from one of the experiments. Only data values above lower limit detection (LLOD) are presented. Na = not applicable, Nd = not determined.

| **Biomarkers** | | **Synovial fluid** | | | **Vendor plasma QC** | | |
| --- | --- | --- | --- | --- | --- | --- | --- |
| Abbreviated protein or gene name | UniProt no | Mean CV, % | CV range, % | N (samples) | Mean CV, % | CV range, % | N (plates) |
| ADA | P00813 | 9.09 | 1.43 - 17.30 | 5 | 4.09 | 1.06 - 14.74 | 19 |
| ARTN | Q5T4W7 | Nd (<LLOD) | Nd (<LLOD) | 0 | 27.97 | Na | 1 |
| AXIN1 | O15169 | 4.30 | 2.97 - 5.40 | 3 | Nd (<LLOD) | Nd (<LLOD) | 0 |
| Beta-NGF | P01138 | 3.83 | Na | 1 | 2.79 | 0.02 - 6.17 | 17 |
| CASP-8 | Q14790 | 6.66 | 2.27 - 10.45 | 5 | 2.25 | 0.24 - 4.48 | 7 |
| CCL4 | P13236 | 6.14 | 0.89 - 9.43 | 5 | 3.71 | 0.10 - 9.90 | 19 |
| CCL19 | Q99731 | 11.06 | 5.21 - 18.28 | 5 | 5.89 | 0.90 - 13.25 | 19 |
| CCL20 | P78556 | 7.00 | 0.27 - 11.93 | 5 | 5.69 | 0.82 - 17.39 | 19 |
| CCL23 | P55773 | 6.59 | 0.63 - 10.49 | 5 | 4.95 | 0.16 - 11.35 | 19 |
| CCL25 | O15444 | 5.36 | 0.61 - 8.06 | 3 | 6.47 | 0.40 - 16.55 | 19 |
| CCL28 | Q9NRJ3 | 10.95 | 1.96 - 17.79 | 5 | 4.08 | 0.91 - 10.53 | 19 |
| CD40 | P25942 | 7.50 | 1.97 - 11.91 | 5 | 4.05 | 0.10 - 12.73 | 19 |
| CDCP1 | Q9H5V8 | 5.93 | 2.68 - 10.39 | 5 | 7.51 | 0.44 - 21.89 | 19 |
| CXCL1 | P09341 | 6.05 | 0.17 - 8.77 | 5 | 4.62 | 0.41 - 12.11 | 19 |
| CXCL5 | P42830 | 8.09 | 4.92 - 13.39 | 5 | 4.95 | 0.48 - 13.48 | 19 |
| CXCL6 | P80162 | 9.29 | 4.56 - 16.37 | 5 | 6.57 | 1.39 - 14.72 | 19 |
| CXCL9 | Q07325 | 5.06 | 0.37 - 10.18 | 5 | 3.69 | 0.14 - 9.40 | 19 |
| CXCL10 | P02778 | 6.82 | 1.32 - 11.39 | 5 | 5.39 | 0.45 - 14.19 | 19 |
| CXCL11 | O14625 | 6.26 | 4.25 - 7.16 | 4 | 3.71 | 0.16 - 12.71 | 19 |
| CST5 | P28325 | 5.66 | 2.82 - 9.45 | 5 | 2.95 | 0.15 - 9.79 | 19 |
| DNER | Q8NFT8 | 5.88 | 1.00 - 9.73 | 5 | 3.56 | 0.01 - 10.39 | 19 |
| CCL11 | P51671 | 4.87 | 2.45 - 7.35 | 5 | 3.81 | 0.07 -11.97 | 19 |
| 4E-BP1 | Q13541 | 8.80 | 4.22 - 13.24 | 5 | 6.06 | 1.37 - 17.98 | 18 |
| FGF-5 | P12034 | 7.72 | Na | 1 | Nd (<LLOD) | Nd (<LLOD) | 0 |
| FGF-19 | O95750 | 7.64 | 3.08 - 10.67 | 5 | 5.34 | 0.16 - 14.49 | 19 |
| FGF-21 | Q9NSA1 | 9.97 | 4.10 - 12.49 | 4 | 3.71 | 0.07 - 9.44 | 19 |
| FGF-23 | Q9GZV9 | Nd (<LLOD) | Nd (<LLOD) | 0 | 3.20 | 0.13 - 5.23 | 3 |
| Flt3L | P49771 | 6.72 | 3.11 - 9.20 | 5 | 4.13 | 0.13 - 15.28 | 19 |
| CX3CL1 | P78423 | 13.17 | 1.50 - 23.04 | 5 | 4.67 | 0.50 - 10.41 | 19 |
| GDNF | P39905 | Nd (<LLOD) | Nd (<LLOD) | 0 | 9.97 | 0.14 - 20.31 | 19 |
| HGF | P14210 | 5.93 | 0.59 - 9.43 | 5 | 3.98 | 0.10 - 12.08 | 19 |
| IFN-gamma | P01579 | 12.54 | 5.09 - 25.00 | 5 | 5.13 | 0.09 - 14.07 | 19 |
| IL-1 alpha | P01583 | Nd (<LLOD) | Nd (<LLOD) | 0 | Nd (<LLOD) | Nd (<LLOD) | 0 |
| IL-2 | P60568 | Nd (<LLOD) | Nd (<LLOD) | 0 | Nd (<LLOD) | Nd (<LLOD) | 0 |
| IL-2RB | P14784 | Nd (<LLOD) | Nd (<LLOD) | 0 | Nd (<LLOD) | Nd (<LLOD) | 0 |
| IL-4 | P05112 | Nd (<LLOD) | Nd (<LLOD) | 0 | 6.13 | 0.27 - 17.23 | 13 |
| IL-5 | P05113 | Nd (<LLOD) | Nd (<LLOD) | 0 | 10.21 | 1.8 - 28.87 | 19 |
| IL-6 | P05231 | 4.78 | 1.67 - 7.82 | 5 | 5.88 | 0.66 - 14.94 | 19 |
| IL-7 | P13232 | 6.72 | 3.30 - 9.70 | 5 | 4.93 | 1.17 -10.45 | 18 |
| IL-8 | P10145 | 7.60 | 4.05 - 10.63 | 5 | 5.44 | 0.17 - 17.63 | 19 |
| IL-10 | P22301 | 9.90 | 7.82 - 11.95 | 3 | 8.04 | 1.68 - 17.23 | 19 |
| IL-10RA | Q13651 | Nd (<LLOD) | Nd (<LLOD) | 0 | 0.68 | 0.48 - 0.87 | 2 |
| IL-10RB | Q08334 | 10.84 | 4.52 - 21.36 | 5 | 5.71 | 0.99 - 14.75 | 19 |
| IL-12B | P29460 | 6.68 | 3.82 - 9.46 | 5 | 3.48 | 0.06 - 7.05 | 19 |
| IL-13 | P35225 | Nd (<LLOD) | Nd (<LLOD) | 0 | 7.44 | 0.35 - 16.04 | 12 |
| IL-15RA | Q13261 | 6.54 | 5.07 - 7.88 | 3 | Nd (<LLOD) | Nd (<LLOD) | 0 |
| IL-17A | Q16552 | Nd (<LLOD) | Nd (<LLOD) | 0 | 6.68 | 0.40 - 16.30 | 19 |
| IL-17C | Q9P0M4 | Nd (<LLOD) | Nd (<LLOD) | 0 | Nd (<LLOD) | Nd (<LLOD) | 0 |
| IL-18 | Q14116 | 5.75 | 2.97 - 10.13 | 5 | 3.69 | 0.13 - 8.07 | 19 |
| IL-18R1 | Q13478 | 6.37 | 0.46 - 11.19 | 5 | 2.96 | 0.19 - 7.62 | 19 |
| IL-20 | Q9NYY1 | Nd (<LLOD) | Nd (<LLOD) | 0 | Nd (<LLOD) | Nd (<LLOD) | 0 |
| IL-20RA | Q9UHF4 | 1.89 | Na | 1 | Nd (<LLOD) | Nd (<LLOD) | 0 |
| IL-22 RA1 | Q8N6P7 | Nd (<LLOD) | Nd (<LLOD) | 0 | Nd (<LLOD) | Nd (<LLOD) | 0 |
| IL-24 | Q13007 | Nd (<LLOD) | Nd (<LLOD) | 0 | Nd (<LLOD) | Nd (<LLOD) | 0 |
| IL-33 | O95760 | 4.47 | 3.23 - 5.90 | 3 | Nd (<LLOD) | Nd (<LLOD) | 0 |
| LAP TGF-beta-1 | P01137 | 7.81 | 4.06 - 14.91 | 5 | 5.08 | 0.85 - 14.23 | 19 |
| LIF | P15018 | 5.83 | 2.10 - 8.58 | 5 | Nd (<LLOD) | Nd (<LLOD) | 0 |
| LIF-R | P42702 | 6.54 | 3.06 - 12.30 | 5 | 4.74 | 0.35 - 13.32 | 19 |
| CSF-1 | P09603 | 5.87 | 2.80 - 8.72 | 5 | 3.88 | 0.17 - 11.49 | 19 |
| CCL3 | P10147 | 4.45 | 0.52 - 8.23 | 5 | 4.48 | 0.15 - 12.43 | 19 |
| MMP-1 | P03956 | 6.77 | 0.43 - 11.57 | 5 | 3.87 | 0.00 - 7.21 | 19 |
| MMP-10 | P09238 | 6.16 | 4.24 - 7.90 | 5 | 3.52 | 0.07 - 11.19 | 19 |
| MCP-1 | P13500 | 5.60 | 1.29 - 7.70 | 5 | 4.25 | 0.24 - 12.59 | 19 |
| MCP-2 | P80075 | 5.90 | 3.47 - 7.72 | 5 | 4.75 | 0.12 - 13.07 | 19 |
| MCP-3 | P80098 | 6.34 | 0.03 - 10.42 | 5 | 5.70 | Na | 1 |
| MCP-4 | Q99616 | 7.65 | 1.72 - 15.74 | 5 | 4.24 | 0.43 - 12.13 | 19 |
| CD244 | Q9BZW8 | 7.87 | 4.09 - 13.05 | 5 | 5.14 | 0.04 - 13.74 | 19 |
| NT-3 | P20783 | Nd (<LLOD) | Nd (<LLOD) | 0 | 6.37 | 0.37 - 16.89 | 19 |
| NRTN | Q99748 | 9.56 | Na | 1 | 7.51 | 0.16 - 23.72 | 19 |
| OSM | P13725 | 6.39 | 3.55 - 10.27 | 5 | 4.46 | 1.07 - 9.42 | 19 |
| OPG | O00300 | 4.63 | 1.42 - 6.28 | 5 | 4.63 | 0.29 - 14.31 | 19 |
| PD-L1 | Q9NZQ7 | 6.90 | 3.66 - 9.90 | 5 | 5.11 | 0.14 - 15.33 | 19 |
| EN-RAGE | P80511 | 8.49 | 4.71 - 15.90 | 5 | 5.81 | 0.12 - 13.81 | 19 |
| SLAMF1 | Q13291 | Nd (<LLOD) | Nd (<LLOD) | 0 | 6.37 | 1.15 - 24.66 | 19 |
| SIRT2 | Q8IXJ6 | 10.80 | 5.43 - 15.82 | 3 | Nd (<LLOD) | Nd (<LLOD) | 0 |
| STAMBP | O95630 | 6.79 | 4.40 - 11.52 | 5 | 3.62 | 0.01 - 13.83 | 19 |
| SCF | P21583 | 6.24 | 1.31 - 10.48 | 5 | 2.53 | 0.27 -7.77 | 19 |
| ST1A1 | P50225 | 10.87 | 2.23 - 18.06 | 5 | Nd (<LLOD) | Nd (<LLOD) | 0 |
| CD5 | P06127 | 9.15 | 1.97 - 18.86 | 5 | 3.71 | 0.09 - 11.76 | 19 |
| CD6 | P30203 | 7.73 | 5.40 - 10.78 | 5 | 5.06 | 0.24 - 13.19 | 19 |
| CD8A | P01732 | 5.27 | 3.88 - 7.40 | 5 | 4.88 | 0.60 - 14.86 | 19 |
| TSLP | Q969D9 | Nd (<LLOD) | Nd (<LLOD) | 0 | 60.22 | Na | 1 |
| TNFB | P01374 | 10.51 | 5.78 - 15.78 | 5 | 4.28 | 0.02 - 10.38 | 19 |
| TRANCE | O14788 | 7.12 | 4.61 - 8.63 | 4 | 5.71 | 1.23 - 17.21 | 19 |
| TRAIL | P50591 | 6.13 | 1.32 - 13.91 | 5 | 4.21 | 0.27 - 9.86 | 19 |
| TGF-alpha | P01135 | 7.53 | 2.15 - 14.64 | 5 | 5.13 | 0.54 -16.05 | 19 |
| TWEAK | O43508 | 8.21 | 6.18 - 10.18 | 5 | 4.64 | 0.10 - 14.30 | 19 |
| TNF | P01375 | 12.52 | 7.33 - 18.82 | 5 | 5.73 | 0.88 - 12.98 | 19 |
| TNFSF14 | O43557 | 6.66 | 2.89 - 9.66 | 3 | 3.70 | 0.02 - 9.69 | 19 |
| TNFRSF9 | Q07011 | 5.73 | 3.93 - 8.94 | 5 | 5.19 | 0.07 - 12.08 | 19 |
| uPA | P00749 | 5.88 | 1.42 - 8.59 | 5 | 4.19 | 0.47 - 13.56 | 19 |
| VEGF-A | P15692 | 4.83 | 0.30 - 7.08 | 5 | 4.19 | 0.35 - 1032 | 19 |

**Table S4**. Assessment of inter plate coefficient of variation (CV) using synovial fluid control sample (n = 4-12 plates), serum quality control (QC) sample (n = 15-19 plates) and the vendor plasma quality QC sample (n = 12-19 plates). Mean CV (%) are presented for biomarkers with data values above lower limit quantification (LLOD). Nd = not determined.

| **Biomarkers** | **UniProt no** | **Synovial fluid** | **Serum QC** | **Vendor plasma QC** |
| --- | --- | --- | --- | --- |
| ADA | P00813 | 28.39 | 6.79 | 8.57 |
| ARTN | Q5T4W7 | Nd (< LLOD) | Nd (< LLOD) | Nd (< LLOD) |
| AXIN1 | O15169 | Nd (< LLOD) | 16.51 | Nd (< LLOD) |
| Beta-NGF | P01138 | Nd (< LLOD) | 11.87 | 7.07 |
| CASP-8 | Q14790 | 15.73 | 13.09 | Nd (< LLOD) |
| CCL4 | P13236 | 13.25 | 11.58 | 13.32 |
| CCL19 | Q99731 | 27.80 | 15.24 | 15.95 |
| CCL20 | P78556 | 25.62 | 19.76 | 12.19 |
| CCL23 | P55773 | 16.83 | 12.28 | 8.46 |
| CCL25 | O15444 | 19.31 | 9.19 | 10.63 |
| CCL28 | Q9NRJ3 | 7.81 | 55.02 | 5.99 |
| CD40 | P25942 | 13.91 | 6.07 | 7.01 |
| CDCP1 | Q9H5V8 | 21.36 | 9.46 | 6.67 |
| CXCL1 | P09341 | 27.46 | 10.60 | 10.27 |
| CXCL5 | P42830 | 21.51 | 12.95 | 12.12 |
| CXCL6 | P80162 | 24.66 | 12.44 | 9.08 |
| CXCL9 | Q07325 | 16.12 | 32.45 | 9.67 |
| CXCL10 | P02778 | 31.00 | 17.59 | 9.69 |
| CXCL11 | O14625 | 19.97 | 57.62 | 10.59 |
| CST5 | P28325 | 10.02 | 6.35 | 7.81 |
| DNER | Q8NFT8 | 14.57 | 5.33 | 7.41 |
| CCL11 | P51671 | 14.65 | 12.97 | 13.65 |
| 4E-BP1 | Q13541 | 34.96 | 92.45 | 79.26 |
| FGF-5 | P12034 | Nd (< LLOD) | Nd (< LLOD) | Nd (< LLOD) |
| FGF-19 | O95750 | 15.15 | 15.12 | 14.30 |
| FGF-21 | Q9NSA1 | 12.86 | 25.11 | 22.25 |
| FGF-23 | Q9GZV9 | Nd (< LLOD) | Nd (< LLOD) | Nd (< LLOD) |
| Flt3L | P49771 | 15.51 | 7.40 | 7.89 |
| CX3CL1 | P78423 | 8.98 | 18.34 | 17.34 |
| GDNF | P39905 | Nd (< LLOD) | 15.28 | 10.61 |
| HGF | P14210 | 19.53 | 7.85 | 8.23 |
| IFN-gamma | P01579 | 25.58 | 24.40 | 11.82 |
| IL-1 alpha | P01583 | Nd (< LLOD) | Nd (< LLOD) | Nd (< LLOD) |
| IL-2 | P60568 | Nd (< LLOD) | Nd (< LLOD) | Nd (< LLOD) |
| IL-2RB | P14784 | Nd (< LLOD) | 9.32 | Nd (< LLOD) |
| IL-4 | P05112 | Nd (< LLOD) | Nd (< LLOD) | 12.35 |
| IL-5 | P05113 | Nd (< LLOD) | 14.43 | 17.12 |
| IL-6 | P05231 | 49.60 | 12.46 | 11.37 |
| IL-7 | P13232 | 7.05 | 10.21 | 10.43 |
| IL-8 | P10145 | 20.24 | 12.19 | 12.23 |
| IL-10 | P22301 | Nd (< LLOD) | 9.63 | 10.50 |
| IL-10RA | Q13651 | Nd (< LLOD) | 9.50 | Nd (< LLOD) |
| IL-10RB | Q08334 | 12.75 | 11.19 | 7.82 |
| IL-12B | P29460 | 16.90 | 10.06 | 8.76 |
| IL-13 | P35225 | Nd (< LLOD) | 11.25 | 7.27 |
| IL-15RA | Q13261 | Nd (< LLOD) | 9.91 | Nd (< LLOD) |
| IL-17A | Q16552 | Nd (< LLOD) | 12.09 | 9.14 |
| IL-17C | Q9P0M4 | Nd (< LLOD) | Nd (< LLOD) | Nd (< LLOD) |
| IL-18 | Q14116 | 11.43 | 8.07 | 7.98 |
| IL-18R1 | Q13478 | 18.35 | 5.64 | 7.00 |
| IL-20 | Q9NYY1 | Nd (< LLOD) | Nd (< LLOD) | Nd (< LLOD) |
| IL-20RA | Q9UHF4 | Nd (< LLOD) | Nd (< LLOD) | Nd (< LLOD) |
| IL-22 RA1 | Q8N6P7 | Nd (< LLOD) | Nd (< LLOD) | Nd (< LLOD) |
| IL-24 | Q13007 | Nd (< LLOD) | 18.53 | Nd (< LLOD) |
| IL-33 | O95760 | 16.21 | Nd (< LLOD) | Nd (< LLOD) |
| LAP TGF-beta-1 | P01137 | 16.49 | 18.19 | 13.97 |
| LIF | P15018 | 29.37 | Nd (< LLOD) | Nd (< LLOD) |
| LIF-R | P42702 | 6.71 | 9.97 | 7.74 |
| CSF-1 | P09603 | 13.91 | 6.63 | 6.40 |
| CCL3 | P10147 | 10.31 | 9.21 | 9.85 |
| MMP-1 | P03956 | 37.13 | 15.55 | 31.85 |
| MMP-10 | P09238 | 9.86 | 10.50 | 10.04 |
| MCP-1 | P13500 | 16.07 | 9.12 | 9.96 |
| MCP-2 | P80075 | 14.49 | 13.50 | 12.87 |
| MCP-3 | P80098 | 29.18 | 9.91 | Nd (< LLOD) |
| MCP-4 | Q99616 | 21.44 | 23.94 | 10.94 |
| CD244 | Q9BZW8 | 13.66 | 6.92 | 8.87 |
| NT-3 | P20783 | Nd (< LLOD) | 9.85 | 9.48 |
| NRTN | Q99748 | Nd (< LLOD) | Nd (< LLOD) | 7.51 |
| OSM | P13725 | 36.18 | 12.94 | 12.85 |
| OPG | O00300 | 13.14 | 8.10 | 5.84 |
| PD-L1 | Q9NZQ7 | 13.44 | 8.59 | 7.28 |
| EN-RAGE | P80511 | 20.05 | 17.04 | 15.83 |
| SLAMF1 | Q13291 | Nd (< LLOD) | 10.44 | 9.65 |
| SIRT2 | Q8IXJ6 | 19.13 | 15.43 | Nd (< LLOD) |
| STAMBP | O95630 | 13.20 | 9.70 | 8.30 |
| SCF | P21583 | 12.74 | 8.73 | 7.34 |
| ST1A1 | P50225 | 25.88 | 31.58 | Nd (< LLOD) |
| CD5 | P06127 | 20.80 | 9.15 | 7.86 |
| CD6 | P30203 | 19.00 | 8.73 | 8.48 |
| CD8A | P01732 | 34.05 | 21.30 | 19.93 |
| TSLP | Q969D9 | Nd (< LLOD) | Nd (< LLOD) | Nd (< LLOD) |
| TNFB | P01374 | 9.71 | 13.10 | 13.46 |
| TRANCE | O14788 | 24.10 | 13.41 | 10.91 |
| TRAIL | P50591 | 17.01 | 13.26 | 11.98 |
| TGF-alpha | P01135 | 13.46 | 10.65 | 12.60 |
| TWEAK | O43508 | 17.32 | 10.32 | 9.00 |
| TNF | P01375 | 24.82 | 27.87 | 29.58 |
| TNFSF14 | O43557 | Nd (< LLOD) | 13.53 | 14.88 |
| TNFRSF9 | Q07011 | 14.55 | 6.84 | 6.37 |
| uPA | P00749 | 21.46 | 6.54 | 7.39 |
| VEGF-A | P15692 | 16.36 | 11.98 | 12.93 |

**Table S5**. Assessment of inter experimental coefficient of variation (CV) using synovial fluid control samples (n = 5-9), serum control samples (n = 12-14) and the Olink (vendor) plasma quality control (QC) sample. Mean CV in % (range) are presented for biomarkers with data values above lower limit quantification (LLOD). The data were either adjusted between the two experiments (using bridging samples) or unadjusted. Nd = not determined.

| **Biomarkers** | | **Synovial fluid** | **Serum** | | **Vendor plasma QC** | |
| --- | --- | --- | --- | --- | --- | --- |
| Abbreviated protein or gene name | UniProt no | Unadjusted | Unadjusted | Adjusted | Unadjusted | Adjusted |
| ADA | P00813 | 66.53 (42.30-108.55) | 51.76 (6.61-84.68) | 17.06 (0.93-46.56) | 51.91 | 0.53 |
| ARTN | Q5T4W7 | Nd (< LLOD) | Nd (< LLOD) | Nd (< LLOD) | Nd (< LLOD) | Nd (< LLOD) |
| AXIN1 | O15169 | Nd (< LLOD) | 19.07 (1.71-66.46) | 14.562 (0.00-54.17) | Nd (< LLOD) | Nd (< LLOD) |
| Beta-NGF | P01138 | Nd (< LLOD) | Nd (< LLOD) | Nd (< LLOD) | Nd (< LLOD) | Nd (< LLOD) |
| CASP-8 | Q14790 | 64.59 (46.60-86.76) | 22.19 (1.98-59.94) | 18.24 (2.42-44.72) | Nd (< LLOD) | Nd (< LLOD) |
| CCL4 | P13236 | 36.06 (15.68-62.61) | 26.36 (11.45-61.11) | 26.40 (8.57-58.73) | 0.41 | 2.48 |
| CCL19 | Q99731 | 40.29 (15.57-71.84) | 20.62 (4.43-58.79) | 19.27 (0.00-62.69) | 7.42 | 2.65 |
| CCL20 | P78556 | 29.26 (14.95-52.97) | 55.84 (23.36-96.61) | 23.94 (0.00-46.99) | 44.33 | 23.15 |
| CCL23 | P55773 | 9.39 (0.50-21.92) | 33.01 (0.17-72.65) | 18.58 (0.00-46.92) | 22.61 | 8.71 |
| CCL25 | O15444 | Nd (< LLOD) | 27.16 (1.15-62.91) | 19.13 (0.00-43.43) | 16.46 | 6.21 |
| CCL28 | Q9NRJ3 | 31.29 (22.64-35.72) | 32.33 (8.66-99.02) | 17.63 (0.00-83.15) | 10.58 | 16.64 |
| CD40 | P25942 | 25.56 (2.20-55.97) | 16.75 (3.25-30.80) | 16.72 (0.00-31.52) | 12.91 | 16.12 |
| CDCP1 | Q9H5V8 | 45.86 (29.05-53.62) | 41.51 (4.46-68.96) | 17.84 (0.00-40.68) | 47.73 | 16.11 |
| CXCL1 | P09341 | 74.48 (46.79-102.68) | 27.46 (7.52-52.63) | 14.96 (0.00-34.07) | 30.06 | 4.90 |
| CXCL5 | P42830 | 24.53 (2.35-49.89) | 19.97 (0.69-49.07) | 16.28 (1.46-35.56) | 11.18 | 3.66 |
| CXCL6 | P80162 | 21.40 (2.48-41.44) | 17.76 (2.58-58.96) | 15.19 (0.00-51.04) | 8.77 | 0.56 |
| CXCL9 | Q07325 | 38.49 (24.47-59.54) | 21.75 (3.67-49.27) | 18.72 (2.96-48.32) | 23.21 | 8.63 |
| CXCL10 | P02778 | 52.20 (6.06-72.46) | 38.36 (12.24-67.29) | 20.04 (0.00-56.45) | 29.99 | 7.63 |
| CXCL11 | O14625 | 26.21 (3.19-60.37) | 25.54 (0.21-61.03) | 21.79 (0.00-51.15) | 20.97 | 8.78 |
| CST5 | P28325 | 54.81 (48.75-58.61) | 48.54 (12.90-132.30) | 31.31 (0.00-126.33) | 25.30 | 11.54 |
| DNER | Q8NFT8 | 18.07 (0.72-31.56) | 21.60 (1.02-44.31) | 14.41 (1.19-35.90) | 24.84 | 4.31 |
| CCL11 | P51671 | 37.26 (21.19-73.49) | 36.20 (3.53-82.71) | 17.10 (0.00-54.58) | 31.54 | 5.07 |
| 4E-BP1 | Q13541 | 37.64 (14.18-76.51) | 42.89 (6.49-81.30) | 44.13 (12.18-86.51) | 23.97 | 34.32 |
| FGF-5 | P12034 | Nd (< LLOD) | Nd (< LLOD) | Nd (< LLOD) | Nd (< LLOD) | Nd (< LLOD) |
| FGF-19 | O95750 | 20.20 (3.46-38.06) | 26.02 (3.29-73.52) | 17.11 (0.37-57.12) | 14.25 | 6.61 |
| FGF-21 | Q9NSA1 | Nd (< LLOD) | 17.12 (0.10-52.16) | 16.80 (0.00-49.20) | 5.67 | 2.29 |
| FGF-23 | Q9GZV9 | Nd (< LLOD) | Nd (< LLOD) | Nd (< LLOD) | Nd (< LLOD) | Nd (< LLOD) |
| Flt3L | P49771 | 17.92 (3.66-30.35) | 17.00 (1.12-50.40) | 16.60 (0.00-43.27) | 0.66 | 7.34 |
| CX3CL1 | P78423 | 39.39 (14.43-56.62) | 71.69 (43.06-96.06) | 14.77 (0.00-38.44) | 90.15 | 22.52 |
| GDNF | P39905 | Nd (< LLOD) | Nd (< LLOD) | Nd (< LLOD) | Nd (< LLOD) | Nd (< LLOD) |
| HGF | P14210 | 30.80 (9.22-44.54) | 25.56 (3.62-49.32) | 17.98 (4.37-50.44) | 26.64 | 6.02 |
| IFN-gamma | P01579 | 40.59 (9.00-63.72) | 20.10 (0.09-71.02) | 20.09 (0.00-71.09) | 5.70 | 5.80 |
| IL-1 alpha | P01583 | Nd (< LLOD) | Nd (< LLOD) | Nd (< LLOD) | Nd (< LLOD) | Nd (< LLOD) |
| IL-2 | P60568 | Nd (< LLOD) | Nd (< LLOD) | Nd (< LLOD) | Nd (< LLOD) | Nd (< LLOD) |
| IL-2RB | P14784 | Nd (< LLOD) | Nd (< LLOD) | Nd (< LLOD) | Nd (< LLOD) | Nd (< LLOD) |
| IL-4 | P05112 | Nd (< LLOD) | Nd (< LLOD) | Nd (< LLOD) | Nd (< LLOD) | Nd (< LLOD) |
| IL-5 | P05113 | Nd (< LLOD) | Nd (< LLOD) | Nd (< LLOD) | 83.55 | 75.45 |
| IL-6 | P05231 | 50.31 (6.64-96.49) | 46.43 (8.05-73.92) | 17.89 (0.00-36.65) | Nd (< LLOD) | Nd (< LLOD) |
| IL-7 | P13232 | 4.04 (0.28-6.98) | 27.51 (5.77-55.50) | 12.34 (0.31-36.18) | Nd (< LLOD) | Nd (< LLOD) |
| IL-8 | P10145 | 59.15 (34.45-84.64) | 27.62 (2.91-50.06) | 19.81 (0.00-38.00) | 27.17 | 8.60 |
| IL-10 | P22301 | Nd (< LLOD) | 15.55 (0.51-46.49) | 14.54 (0.00-52.94) | 17.14 | 9.84 |
| IL-10RA | Q13651 | Nd (< LLOD) | Nd (< LLOD) | Nd (< LLOD) | Nd (< LLOD) | Nd (< LLOD) |
| IL-10RB | Q08334 | 33.02 (19.92-57.15) | 17.12 (1.64-44.80) | 14.40 (0.11-36.43) | 5.40 | 3.73 |
| IL-12B | P29460 | 56.00 (40.01-81.39) | 17.42 (0.05-47.60) | 17.41 (0.00-47.64) | 9.56 | 9.52 |
| IL-13 | P35225 | Nd (< LLOD) | Nd (< LLOD) | Nd (< LLOD) | Nd (< LLOD) | Nd (< LLOD) |
| IL-15RA | Q13261 | Nd (< LLOD) | 30.55 (5.83-49.73) | 10.26 (0.00-26.13) | Nd (< LLOD) | Nd (< LLOD) |
| IL-17A | Q16552 | Nd (< LLOD) | 34.21 (12.00-82.41) | 16.69 (0.00-82.41) | Nd (< LLOD) | Nd (< LLOD) |
| IL-17C | Q9P0M4 | Nd (< LLOD) | Nd (< LLOD) | Nd (< LLOD) | Nd (< LLOD) | Nd (< LLOD) |
| IL-18 | Q14116 | 16.44 (2.88-49.08) | 36.42 (6.42-80.14) | 20.59 (0.00-47.47) | 27.23 | 13.88 |
| IL-18R1 | Q13478 | 21.48 (2.38-48.90) | 15.91 (0.02-35.84) | 15.92 (0.00-30.43) | 3.67 | 9.39 |
| IL-20 | Q9NYY1 | Nd (< LLOD) | Nd (< LLOD) | Nd (< LLOD) | Nd (< LLOD) | Nd (< LLOD) |
| IL-20RA | Q9UHF4 | Nd (< LLOD) | Nd (< LLOD) | Nd (< LLOD) | Nd (< LLOD) | Nd (< LLOD) |
| IL-22 RA1 | Q8N6P7 | Nd (< LLOD) | Nd (< LLOD) | Nd (< LLOD) | Nd (< LLOD) | Nd (< LLOD) |
| IL-24 | Q13007 | Nd (< LLOD) | Nd (< LLOD) | Nd (< LLOD) | Nd (< LLOD) | Nd (< LLOD) |
| IL-33 | O95760 | Nd (< LLOD) | Nd (< LLOD) | Nd (< LLOD) | Nd (< LLOD) | Nd (< LLOD) |
| LAP TGF-beta-1 | P01137 | 24.95 (4.20-60.57) | 36.64 (6.52-64.86) | 19.46 (0.00-60.68) | 43.91 | 10.92 |
| LIF | P15018 | 21.58 (8.27-33.87) | Nd (< LLOD) | Nd (< LLOD) | Nd (< LLOD) | Nd (< LLOD) |
| LIF-R | P42702 | 19.99 (12.69-30.12) | 19.98 (0.48-58.75) | 15.72 (4.12-45.37) | 15.35 | 0.08 |
| CSF-1 | P09603 | 35.32 (22.95-55.01) | 11.88 (0.28-30.74) | 11.88 (0.95-25.86) | 4.87 | 11.58 |
| CCL3 | P10147 | 63.24 (43.38-83.95) | 22.31 (5.54-53.13) | 22.19 (2.87-61.63) | 6.23 | 3.95 |
| MMP-1 | P03956 | 24.17 (1.80-84.56) | 125.32 (100.33-133.96) | 25.49 (0.43-86.78) | 130.31 | 0.36 |
| MMP-10 | P09238 | 70.71 (56.10-86.37) | 105.07 (95.87-120.50) | 12.01 (0.00-42.17) | 104.46 | 1.22 |
| MCP-1 | P13500 | 7.41 (0.57-19.50) | 23.99 (1.12-59.15) | 17.63 (1.49-41.09) | 17.28 | 10.84 |
| MCP-2 | P80075 | 56.14 (39.17-73.54) | 24.46 (2.07-43.82) | 18.01 (0.00-58.08) | 22.51 | 4.15 |
| MCP-3 | P80098 | 22.41 (0.93-44.17) | 14.31 (0.60-34.79) | 14.17 (0.00-35.35) | Nd (< LLOD) | Nd (< LLOD) |
| MCP-4 | Q99616 | 48.09 (24.57-72.03) | 24.05 (1.00-58.86) | 20.65 (0.00-38.99) | 15.90 | 6.66 |
| CD244 | Q9BZW8 | 14.58 (1.45-50.31) | 22.25 (0.03-46.39) | 15.07 (0.00-33.51) | 24.82 | 11.04 |
| NT-3 | P20783 | Nd (< LLOD) | Nd (< LLOD) | Nd (< LLOD) | 20.67 | 11.53 |
| NRTN | Q99748 | Nd (< LLOD) | Nd (< LLOD) | Nd (< LLOD) | 5.49 | 11.13 |
| OSM | P13725 | 37.27 (4.21-82.25) | 19.01 (1.63-40.17) | 19.03 (0.00-36.24) | 2.16 | 2.07 |
| OPG | O00300 | 19.21 (8.85-33.23) | 17.39 (0.99-52.53) | 15.21 (0.00-41.72) | 6.02 | 6.14 |
| PD-L1 | Q9NZQ7 | 29.01 (8.82-49.97) | 47.10 (6.92-65.92) | 11.27 (0.00-39.66) | 48.53 | 2.90 |
| EN-RAGE | P80511 | 45.76 (16.39-66.76) | 30.50 (3.24-102.22) | 27.69 (0.00-106.56) | 9.21 | 18.68 |
| SLAMF1 | Q13291 | Nd (< LLOD) | 14.50 (0.20-39.57) | 10.72 (0.00-28.94) | 21.63 | 10.47 |
| SIRT2 | Q8IXJ6 | 28.81 (8.83-51.76) | 29.77 (2.16-53.97) | 19.84 (0.00-72.75) | Nd (< LLOD) | Nd (< LLOD) |
| STAMBP | O95630 | 22.78 (1.88-57.73) | 16.13 (0.30-34.55) | 15.15 (5.28-42.75) | 14.49 | 5.68 |
| SCF | P21583 | 50.05 (31.07-78.82) | 14.50 (3.97-35.48) | 13.35 (0.00-39.17) | 11.31 | 7.36 |
| ST1A1 | P50225 | 15.15 (3.96-37.26) | 19.06 (3.27-48.12) | 15.37 (0.00-37.77) | Nd (< LLOD) | Nd (< LLOD) |
| CD5 | P06127 | 16.85 (0.63-67.65) | 49.37 (32.36-73.32) | 12.87 (0.00-30.33) | 38.84 | 10.51 |
| CD6 | P30203 | 24.69 (8.73-60.47) | 53.41 (21.06-86.86) | 16.10 (0.00-45.19) | 45.29 | 7.43 |
| CD8A | P01732 | 44.69 (4.79-114.59) | 23.56 (2.05-69.38) | 23.48 (0.00-75.83) | 17.38 | 8.70 |
| TSLP | Q969D9 | Nd (< LLOD) | Nd (< LLOD) | Nd (< LLOD) | Nd (< LLOD) | Nd (< LLOD) |
| TNFB | P01374 | 15.36 (3.19-28.00) | 12.87 (0.76-56.98) | 12.75 (0.00-57.62) | 0.07 | 0.69 |
| TRANCE | O14788 | 28.75 (13.53-57.79) | 14.94 (0.99-51.10) | 14.97 (0.75-51.32) | 6.46 | 6.21 |
| TRAIL | P50591 | 43.97 (7.29-76.20) | 37.72 (1.38-72.00) | 14.76 (1.92-53.52) | 44.39 | 6.27 |
| TGF-alpha | P01135 | 54.72 (28.85-72.18) | 45.54 (5.79-72.51) | 16.65 (0.00-40.91) | 45.13 | 1.14 |
| TWEAK | O43508 | 27.56 (7.55-59.43) | 28.23 (7.48-56.33) | 18.84 (0.20-57.74) | 29.58 | 4.93 |
| TNF | P01375 | 13.53 (7.62-20.94) | 20.13 (3.32-61.92) | 19.11 (0.31-58.57) | 2.43 | 1.65 |
| TNFSF14 | O43557 | Nd (< LLOD) | 24.37 (1.09-53.04) | 17.91 (0.00-43.00) | 17.71 | 9.68 |
| TNFRSF9 | Q07011 | 8.81 (1.02-17.11) | 14.57 (1.96-44.90) | 14.08 (0.00-41.51) | 2.86 | 6.59 |
| uPA | P00749 | 7.82 (0.70-27.68) | 17.32 (1.06-48.32) | 13.96 (0.47-39.85) | 4.29 | 5.09 |
| VEGF-A | P15692 | 11.28 (0.13-27.69) | 47.99 (24.11-81.79) | 17.75 (0.12-38.03) | 46.46 | 6.09 |

**Table S6**. Assessment of dilution linearity of nine synovial fluid control samples. Amount of samples where the biomarkers fulfilled the selection criteria (values above lower limit of detection, coefficient of variation of linearity <21% and recovery between 74-127%) are shown (i.e., n = 1-9 samples). Biomarkers marked in *italics* did not fulfill the selection criteria in any of the nine control samples (i.e., n = 0 samples). In total 61 biomarkers fulfilled the selection criteria in any of the nine synovial fluid control samples.

| **Biomarkers** | **UniProt no** | **Samples, n** |
| --- | --- | --- |
| *ADA* | *P00813* | *0* |
| *ARTN* | *Q5T4W7* | *0* |
| *AXIN1* | *O15169* | *0* |
| *Beta-NGF* | *P01138* | *0* |
| CASP-8 | Q14790 | 9 |
| CCL4 | P13236 | 8 |
| CCL19 | Q99731 | 3 |
| CCL20 | P78556 | 7 |
| CCL23 | P55773 | 4 |
| CCL25 | O15444 | 2 |
| *CCL28* | *Q9NRJ3* | *0* |
| CD40 | P25942 | 6 |
| CDCP1 | Q9H5V8 | 5 |
| CXCL1 | P09341 | 8 |
| CXCL5 | P42830 | 9 |
| CXCL6 | P80162 | 6 |
| CXCL9 | Q07325 | 5 |
| CXCL10 | P02778 | 5 |
| CXCL11 | O14625 | 8 |
| CST5 | P28325 | 3 |
| DNER | Q8NFT8 | 8 |
| CCL11 | P51671 | 4 |
| 4E-BP1 | Q13541 | 3 |
| *FGF-5* | *P12034* | *0* |
| FGF-19 | O95750 | 9 |
| FGF-21 | Q9NSA1 | 1 |
| *FGF-23* | *Q9GZV9* | *0* |
| Flt3L | P49771 | 7 |
| CX3CL1 | P78423 | 5 |
| *GDNF* | *P39905* | *0* |
| HGF | P14210 | 6 |
| IFN-gamma | P01579 | 4 |
| *IL-1 alpha* | *P01583* | *0* |
| *IL-2* | *P60568* | *0* |
| *IL-2RB* | *P14784* | *0* |
| *IL-4* | *P05112* | *0* |
| *IL-5* | *P05113* | *0* |
| IL-6 | P05231 | 6 |
| *IL-7* | *P13232* | *0* |
| IL-8 | P10145 | 9 |
| IL-10 | P22301 | 3 |
| *IL-10RA* | *Q13651* | *0* |
| IL-10RB | Q08334 | 5 |
| IL-12B | P29460 | 6 |
| *IL-13* | *P35225* | *0* |
| *IL-15RA* | *Q13261* | *0* |
| *IL-17A* | *Q16552* | *0* |
| *IL-17C* | *Q9P0M4* | *0* |
| IL-18 | Q14116 | 6 |
| IL-18R1 | Q13478 | 6 |
| *IL-20* | *Q9NYY1* | *0* |
| *IL-20RA* | *Q9UHF4* | *0* |
| *IL-22 RA1* | *Q8N6P7* | *0* |
| *IL-24* | *Q13007* | *0* |
| *IL-33* | *O95760* | *0* |
| LAP TGF-beta-1 | P01137 | 3 |
| LIF | P15018 | 4 |
| *LIF-R* | *P42702* | *0* |
| CSF-1 | P09603 | 6 |
| CCL3 | P10147 | 7 |
| MMP-1 | P03956 | 1 |
| MMP-10 | P09238 | 4 |
| MCP-1 | P13500 | 8 |
| MCP-2 | P80075 | 8 |
| MCP-3 | P80098 | 4 |
| MCP-4 | Q99616 | 9 |
| CD244 | Q9BZW8 | 5 |
| *NT-3* | *P20783* | *0* |
| *NRTN* | *Q99748* | *0* |
| OSM | P13725 | 8 |
| OPG | O00300 | 5 |
| PD-L1 | Q9NZQ7 | 5 |
| EN-RAGE | P80511 | 4 |
| *SLAMF1* | *Q13291* | *0* |
| SIRT2 | Q8IXJ6 | 2 |
| STAMBP | O95630 | 6 |
| SCF | P21583 | 8 |
| *ST1A1* | *P50225* | *0* |
| CD5 | P06127 | 5 |
| CD6 | P30203 | 3 |
| CD8A | P01732 | 5 |
| *TSLP* | *Q969D9* | *0* |
| TNFB | P01374 | 1 |
| TRANCE | O14788 | 1 |
| TRAIL | P50591 | 3 |
| TGF-alpha | P01135 | 1 |
| TWEAK | O43508 | 9 |
| *TNF* | *P01375* | *0* |
| TNFSF14 | O43557 | 2 |
| TNFRSF9 | Q07011 | 6 |
| uPA | P00749 | 7 |
| VEGF-A | P15692 | 9 |

**Table S7**. Assessment of dilution linearity of nine synovial control samples (A-I). The number of biomarkers fulfilling the selection criteria (values above lower limit of detection, coefficient of variation of linearity <21% and recovery between 74-127%) and the recovery (in CV%) are shown for each control sample.

| **Samples A-D** |  |  |  |  |  |  |  |  |
| --- | --- | --- | --- | --- | --- | --- | --- | --- |
| Samples | A |  | B |  | C |  | D |  |
| Dilutions, range (span) | 1-25x (25x) | 2-25x (12.5x) | 1-25x (25x) | 2-25x (12.5x) | 1-25x (25x) | 2-25x (12.5x) | 1-25x (25x) | 2-25x (12.5x) |
| Biomarkers, n | 19 | 35 | 26 | 41 | 26 | 32 | 36 | 44 |
| CV% of NPX, mean (range) | 13.4 (9.7-17.7) | 10.8 (3.6-16.3) | 12.0 (4.6-19.3) | 10.3 (2.4-20.1) | 9.2 (1.7-17.9) | 9.1 (1.4-19.0) | 12.6 (2.6-20.0) | 12.0 (1.3-19.7) |
|  |  |  |  |  |  |  |  |  |
| **Samples E-G** |  |  |  |  |  |  |  |  |
| Samples | E |  | F |  | G |  |  |  |
| Dilutions, range (span) | 1-10x (10x) | 2-10x (5x) | 1-10x (10x) | 2-10x (5x) | 1-10x (10x) | 2-10x (5x) |  |  |
| Biomarkers, n | 48 | 51 | 43 | 45 | 42 | 42 |  |  |
| CV% of NPX, mean (range) | 10.0 (2.6-20.2) | 9.4 (2.1-18.6) | 9.7 (2.5-19.2) | 9.9 (2.3-19.3) | 12.9 (6.3-19.4) | 13.3 (4.2-19.7) |  |  |
|  |  |  |  |  |  |  |  |  |
| **Samples H, G** |  |  |  |  |  |  |  |  |
| Samples | H | I |  |  |  |  |  |  |
| Dilutions, range (span) | 1-4x (4x) | 1-4x (4x) |  |  |  |  |  |  |
| Biomarkers, n | 44 | 44 |  |  |  |  |  |  |
| CV% of NPX, mean (range) | 12.4 (6.4-20.3) | 15.5 (10.2-20.8) |  |  |  |  |  |  |

NPX = normalized protein expression
