# Supplementary material for: Technical performance of a proximity extension assay inflammation biomarker panel with synovial fluid
Source: Osteoarthr Cartil Open. 2022 Jul 7;4(3):100293. doi: 10.1016/j.ocarto.2022.100293 (PMC9718077; doi:10.1016/j.ocarto.2022.100293)
Supplement: Multimedia component 4 [file mmc4.docx]

**Table S10.** Pearson correlation between cytokine concentrations measured by proximity extension assay (PEA) and Meso Scale Discovery (MSD) measurements.

|  |  | N (%> LLOD) | r (95% CI) | P-values |
| --- | --- | --- | --- | --- |
| **Synovial fluid** |  |  |  |  |
|  | IL-6 | 232 (87.5) | 0.943 (0.926, 0.955) | <0.001 |
|  | IL-8 | 310 (100) | 0.923 (0.905, 0.938) | <0.001 |
|  | IL-10 | 143 (48.3) | 0.891 (0.851, 0.921) | <0.001 |
|  | IFN-γ | 80 (71.3) | 0.281 (0.065, 0.471) | 0.012 |
|  | TNF | 299 (81.3) | 0.686 (0.621, 0.742) | <0.001 |
| **Serum** |  |  |  |  |
|  | IL-6 | 110 (100) | 0.265 (0.081, 0.430) | 0.005 |
|  | IL-8 | 546 (100) | 0.909 (0.894, 0.923) | <0.001 |
|  | IL-10 | 256 (99.6%) | -0.092 (-0.212, 0.031) | 0.144 |
|  | IFN-γ | 104 (100) | 0.753 (0.655, 0.826) | <0.001 |
|  | TNF | 546 (100) | 0.263 (0.184, 0.340) | <0.001 |

LLOD = lower limit of detection, CI = confidence interval.

**Table S11.** Proportional bias between proximity extension assay (PEA) and Meso Scale Discovery (MSD). To estimate the effect of proportional bias between the assays, we created a High and a Low concentration group for each of the five cytokines based on their concentration measured by MSD. For each biomarker, samples with concentration in the 10^th^ percentile were selected as Low, and samples in the 90^th^ percentile as High. The mean concentration in groups High and Low was calculated for MSD and PEA measurements, respectively, and the ratio in means High to Low was used to estimate the effect of proportional bias between assay measurements.

|  |  | MSD  (Ratio High/Low) | PEA  (Ratio High/Low) | Fold higher Ratio High/Low by MSD compared to PEA |
| --- | --- | --- | --- | --- |
| **Synovial fluid** |  |  |  |  |
|  | IL-6 | 10317 | 2105 | 4.9 |
|  | IL-8 | 53 | 55 | 1.0 |
|  | IL-10 | 27 | 8.9 | 3.0 |
|  | IFN-γ | 60 | 37 | 1.6 |
|  | TNF | 8.5 | 2.3 | 3.7 |
| **Serum** |  |  |  |  |
|  | IL-6 | 12 | 3.2 | 3.6 |
|  | IL-8 | 11 | 6.8 | 1.6 |
|  | IL-10 | 77 | 0.9 | 86 |
|  | IFN-γ | 16 | 18 | 0.9 |
|  | TNF | 3.4 | 1.4 | 2.4 |
